# Loss of O-GlcNAcylation modulates mTORC1 and autophagy in **β** cells, driving diabetes 2 progression

**DOI:** 10.1172/jci.insight.183033

**Published:** 2024-12-06

**Authors:** Seokwon Jo, Nicholas Esch, Anh Nguyen, Alicia Wong, Ramkumar Mohan, Clara Kim, Manuel Blandino-Rosano, Ernesto Bernal-Mizrachi, Emilyn U. Alejandro

**Affiliations:** 1 Department of Integrative Biology & Physiology, University of Minnesota Medical School, Minneapolis, Minnesota, USA.; 2 Department of Internal Medicine, Division of Endocrinology, Metabolism and Diabetes, Miller School of Medicine, University of Miami, Miami, Florida, USA.

**Keywords:** Endocrinology, Metabolism, Autophagy, Beta cells, Diabetes

## Abstract

Type 2 diabetes (T2D) arises when pancreatic β cells fail to produce sufficient insulin to control blood glucose appropriately. Aberrant nutrient sensing by O-GlcNAcylation and mTORC1 is linked to T2D and the failure of insulin-producing β cells. However, the nature of their crosstalk in β cells remains unexplored. Recently, O-GlcNAcylation, a posttranslation modification controlled by enzymes O-GlcNAc transferase/O-GlcNAcase (OGT/OGA), emerged as a pivotal regulator for β cell health; deficiency in either enzyme causes β cell failure. The present study investigates the previously unidentified connection between nutrient sensor OGT and mTORC1 crosstalk to regulate β cell mass and function in vivo. We show reduced OGT and mTORC1 activity in islets of a preclinical β cell dysfunction model and islets from humans with obesity. Using loss or gain of function of OGT, we identified that O-GlcNAcylation positively regulated mTORC1 signaling in β cells. O-GlcNAcylation negatively modulated autophagy, as the removal of OGT increased autophagy, while the deletion of OGA decreased it. Increasing mTORC1 signaling, via deletion of TSC2, alleviated the diabetic phenotypes by increasing β cell mass but not β cell function in OGT-deficient mice. Downstream phospho-protein signaling analyses revealed diverging effects on MKK4 and calmodulin signaling between islets with OGT, TSC2, or combined deletion. These data provide evidence of OGT’s significance as an upstream regulator of mTORC1 and autophagy, crucial for the regulation of β cell function and glucose homeostasis.

## Abstract 2

Post-translational modification crosstalk through autophagy in pancreatic beta-cell is critical for development of diabetes.

## Graphical abstract

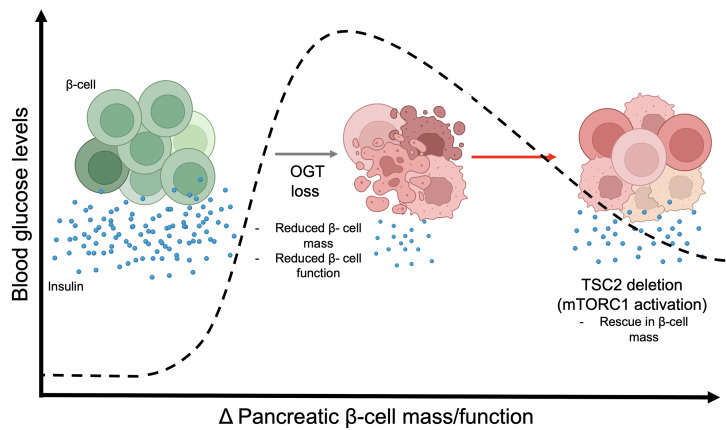

## Introduction

Type 2 diabetes (T2D) affects over 500 million individuals worldwide and imposes great burdens on patients and society. The central pathology of diabetes is the dysregulation of glucose homeostasis that arises from malfunctions in multiple processes from uptake and storage of glucose inside cells (1). However, the determining factor that tips the balance toward the development of T2D is the insufficiency of insulin action, an anabolic hormone released from pancreatic β cells to stimulate glucose uptake into peripheral tissues. Persistent diabetes is linked to various complications such as cardiovascular disease, neuropathy, nephropathy, retinopathy, and stroke (2), making it a life-threatening condition.

Mechanistic Target of Rapamycin Complex 1 (mTORC1) signaling is a major nutrient-sensitive signaling pathway that modulates cell growth and protective cellular responses such as autophagy, a regulated process in which proteins and organelles are targeted for degradation, to maintain a good quality pool of organelles and proteins inside the cell (3). Under nutrient stress such as fasting, a proper level of autophagy is necessary for appropriate cellular energetics to maintain insulin secretion and cell viability. Presently, our understanding of the direct connection between nutrient excess and autophagy, particularly upstream of mTORC1 signaling, remains unclear.

Recent studies show the critical role of nutrient- and stress-sensitive intracellular posttranslational modification, O-GlcNAcylation, that governs and modulates cell signaling nodes that affect β cell mass and function. O-GlcNAcylation is a dynamic posttranslational modification whose substrate is derived from glucose, lipids, and amino acids. This process is maintained by 2 enzymes, O-GlcNAc transferase (OGT) to add and O-GlcNAcase (OGA) to remove the O-GlcNAc moiety on serine/threonine of target proteins, affecting their function, localization, and stability (4). The homeostatic balance between OGT and OGA cycling integrates the nutrient status of the cell with intracellular responses to regulate metabolism and glucose homeostasis. Within the pancreas, OGT is highly expressed in endocrine islets (5) and has key modulatory properties that orchestrate β cell health and function in response to nutrient stress and insulin demand (5). β Cell–specific deletion of OGT causes diabetes in part by reduced β cell mass and insulin secretion associated with a decreased number of insulin granules and mitochondrial dysfunction (6, 7). O-GlcNAcylation is also required for insulin hypersecretion in early adaptive stages of obesity. Thus, in β cells, O-GlcNAcylation promotes positive regulatory effects on β cell health (growth, survival, and function). As a nutrient- and stress-sensor protein, the effects of OGT on autophagy in β cells remain unexplored.

OGT and mTORC1 signaling are dysregulated in many diseases including T2D. Although we have made advancements in our understanding of the roles of nutrient-sensing mTORC1 and OGT by studying them independently, how they crosstalk to affect β cell function and glucose homeostasis in vivo is untested. In this study, we hypothesize that OGT elicits crosstalk with mTORC1 to regulate β cell mass and function homeostasis. We utilized both in vitro and in vivo approaches with loss or gain of function of OGT or mTORC1, to elucidate the relationship between O-GlcNAcylation and mTORC1 and autophagy-mediated β cell mass and function.

## Results

### Dysregulation of OGT and mTORC1 in models of β cell dysfunction in mice and humans.

**Figure 1:**
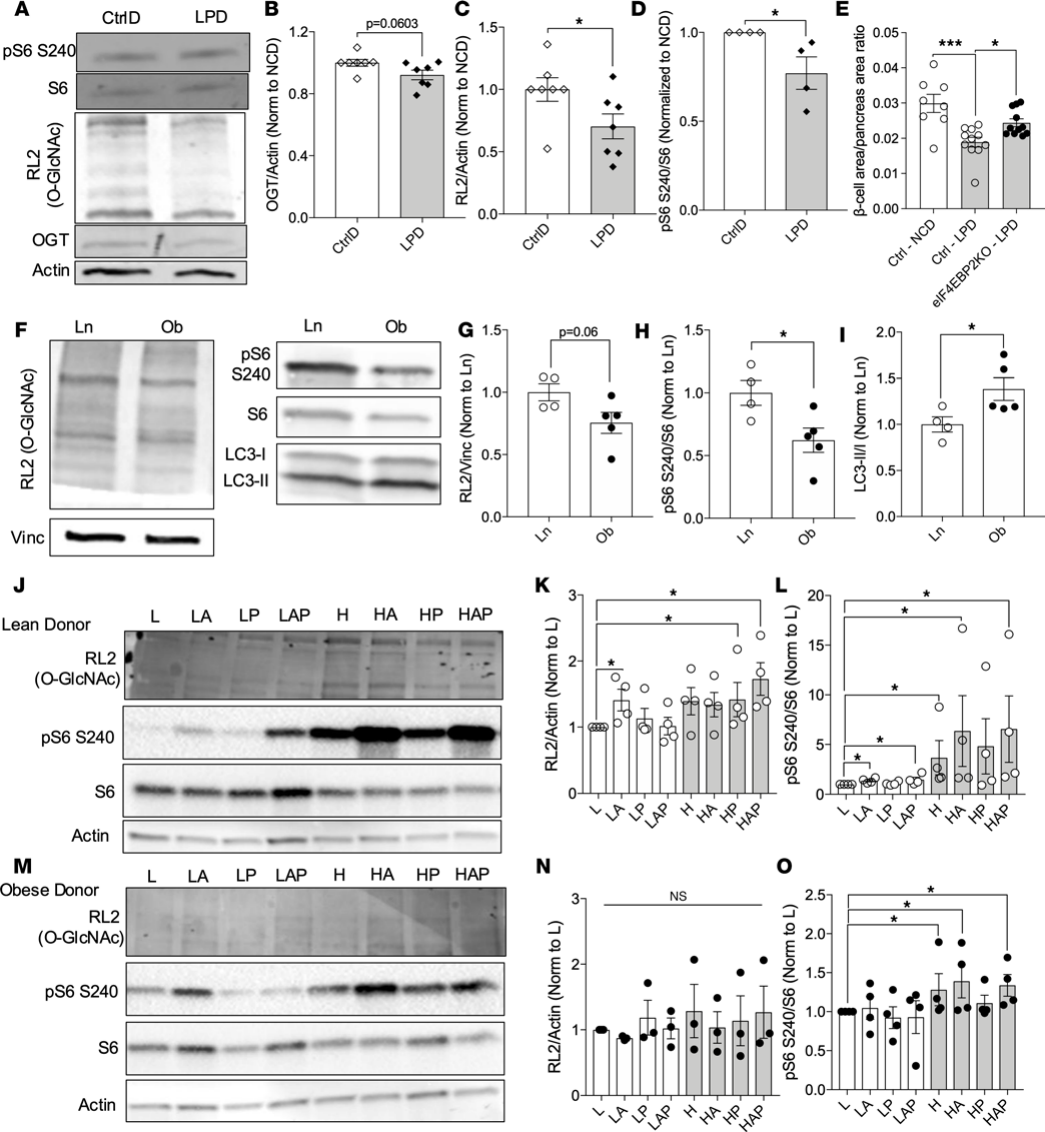
Aberrant mTORC1 and O-GlcNAcylation signaling in models of islet stress. (**A**–**D**) OGT, pS6 S240, S6, and O-GlcNAcylation (RL2 antibody) levels from WT mouse islets from the offspring born to dam fed control diet (CtrlD) or low protein diet during pregnancy (LPD) (*n* = 4–7). (**E**) β Cell area to pancreas area ratio from control and eIF4EBP2-KO (BP2-KO) neonatal mice born to either dam fed CtrlD or low protein diet (*n* = 8–11). (**F**–**I**) Representative immunoblot and analysis of pS6 S240, S6, RL2 (pan-O-GlcNAc), and LC3 from islets from patients who are lean or have obesity (*n* = 4–5). (**J**–**O**) Islets from patients who are lean or have obesity were treated with low or high glucose (L, 3 mM; H, 16.7 mM), amino acid (A), and/or palmitate/BSA (P; 16:4; 100 μM; palmitate [Palm]) for 6 hours and assessed in immunoblot for pS6 S240, S6 and RL2 (pan-O-GlcNAc) (*n* = 3–4 donors). One of 4 obese donor islets did not show any RL2 signaling. Statistical analysis by Student’s *t* test (**B**–**E**, and **G**–**I**) and 1-way ANOVA (**K**, **L**, **N**, and **O**). *P ≤ 0.05, ***P < 0.001.

β Cell mass and function are affected by both genetic and environmental factors (e.g., fetal-origins predisposition of T2D due to β cell dysfunction; ref. 8) in part by reducing key proteins such as Pdx1 and mTOR (9). For example, the offspring of dams fed a low-protein diet (LPD) throughout pregnancy (a model of maternal malnutrition) (9) or individuals born during the Dutch famine (ca. 1944–1945) have increased susceptibility to T2D (10). Islets of LPD offspring exhibited β cell dysfunction and mechanistically, in part, through microRNAs alterations targeting mTOR(9). Here, we report that OGT and its activity, O-GlcNAcylation (assessed by using RL2 ]pan-O-GlcNAc] antibody), are also reduced in LPD islets (Figure 1, A–C), in addition to loss of mTORC1 signaling (Figure 1D). OGA was reduced, and no changes in total S6 were observed between control diet (CtrlD) and LPD (Supplemental Figure 1, A–C; supplemental material available online with this article; https://doi.org/10.1172/jci.insight.183033DS1). A downstream arm of the mTORC1 pathway is the phosphorylation and inactivation of eIF4EBP to increase protein translation. Deletion of mTOR target eIF4EBP2 (eIF4EBP2-KO) protected loss of β cell area in LPD offspring (Figure 1E), showing mechanistically the importance of mTORC1-eIF4EBP2 in maintaining β cell health.

Obesity is a risk factor for T2D and is associated with β cell dysfunction. In islets from patients who are lean or have obesity, we analyzed the baseline protein levels of OGT and mTORC1, along with their activity. We previously reported that donors with obesity exhibited lower O-GlcNAcylation (11). In this new cohort of human islet samples, we show that the reduction O-GlcNAcylation seen in islets from patients with obesity was associated with lower mTORC1 activity, measured by the phosphorylation of the downstream target, S6 at Ser240 (Figure 1, F–H). We observed comparable values in the total protein levels of OGT, OGA, and mTOR (Supplemental Figure 1, D–G) among these islets. Total S6 protein was, however, reduced significantly in islets from patients with obesity versus patients who are lean (Supplemental Figure 1H). During the early phase of obesity in mice (6 weeks in HFD treatment), O-GlcNAcylation increases. However, following sustained obesity (18 weeks after HFD), OGT protein level decreases below normal levels (11). mTORC1 regulates a myriad of cellular signaling pathways, including inhibition of autophagy. With lower mTORC1 activity, we observed higher autophagy, show by increased LC3, a marker of autophagy (lipidation in response to autophagosome maturation) (Figure 1, F and I). Next, we assessed the human donor islets’ ability to engage nutrient sensor protein activities, in response to increased nutrient signals from glucose, amino acid, and lipids. In lean donor islets, we observed a 1.3-fold increase in mTORC1 activity with amino acid and a 6-fold increase with glucose stimulation (Figure 1, J and L). This change in phospho-S6 was without any changes to the total level of S6 protein (Supplemental Figure 1I). Concurrent analysis of RL2 in the same samples revealed a 1.4-fold increase in O-GlcNAcylation with amino acid and a 1.7-fold increase with glucose treatment (Figure 1, J and K). However, in obese donor islets, amino acids did not alter mTORC1 activity, but a 1.3-fold increase was observed in response to glucose (Figure 1, M and O). No alterations to O-GlcNAcylation levels in response to amino acids and glucose in obese donor islets (Figure 1, M and N). Palmitate alone had no effect on mTORC1 activity or O-GlcNAcylation level in either lean or obese donor islets. Altogether, these preliminary data demonstrate a positive correlation between OGT and mTORC1 activity in islets from individuals who are lean in response to environmental stressors, whereas this response is altered in islets from patients with obesity.

### OGT positively regulates mTORC1 activity in primary β cells.

**Figure 2:**
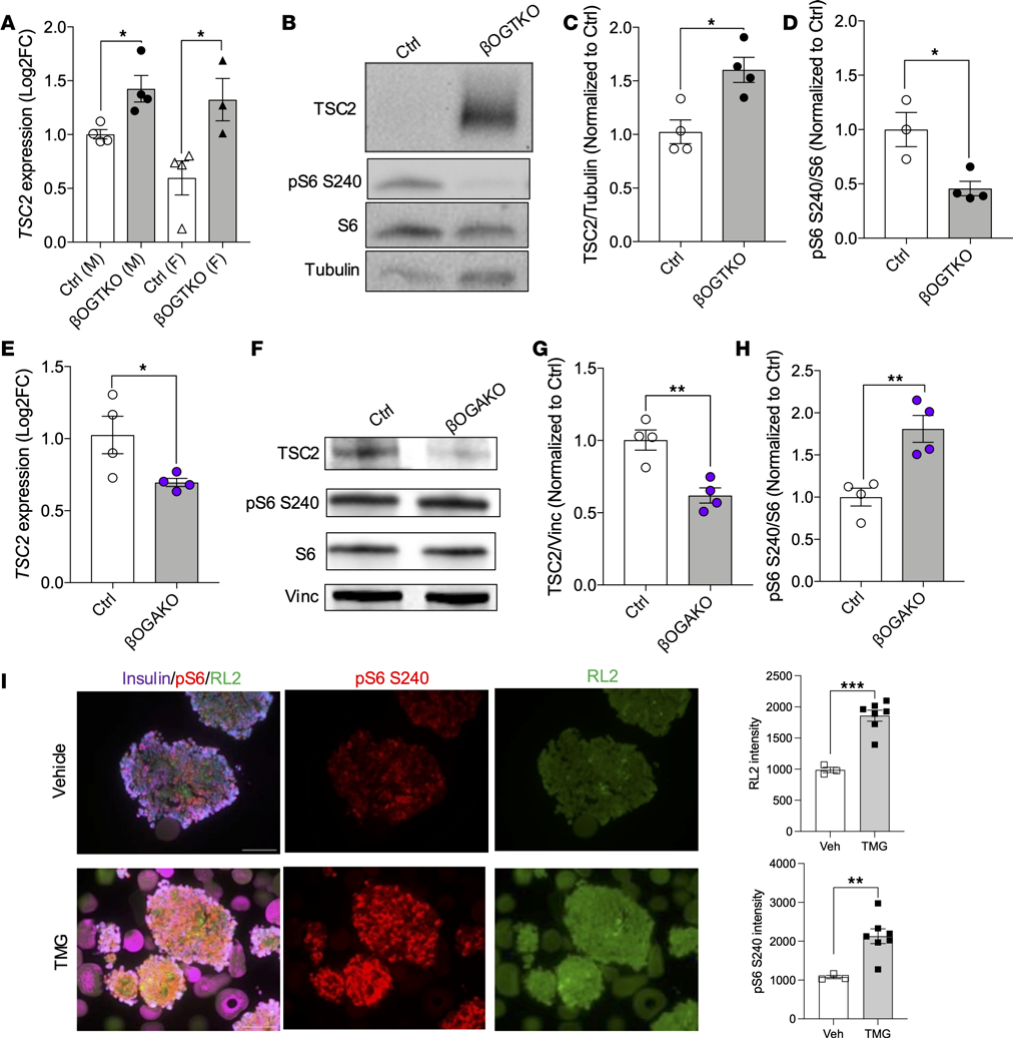
OGT modulates mTORC1 signaling in pancreatic β cells. (**A**) qPCR analysis of *Tsc2* mRNA from male and female, control and βOGT-KO islets; normalized to 36B4 (*n* = 3-4). (**B**–**D**) Representative immunoblot and analysis of TSC2, pS6 S240, and S6 from control and βOGT-KO islets (*n* = 3-4). (**E**) Quantatitative PCR analysis of *Tsc2* mRNA from male control and βOGA-KO islets; normalized to 36B4 (*n* = 4). (**F**–**H**) Representative immunoblot and analysis of TSC2 and pS6 S240 from control and βOGA-KO islets (*n* = 3–5). (**I**) Representative immunofluorescence imaging and quantification of insulin, pS6 S240, and RL2 from histogel embedded human donor (lean) islets treated with vehicle/control or TMG (30 μM) for 12 hours. Magnification, ×600. Scale bar: 100 μm. Statistical analysis by Student’s *t* test. *P ≤ 0.05, **P < 0.01, ***P < 0.001.

To delineate the potential relationship between OGT and mTOR, we performed RNA-Seq and proteomics on islets from β cell–specific OGT-deficient mice (βOGT-KO) (7, 11). Both transcriptomics and proteomics analysis from primary islets of βOGT-KO show TSC2, a negative regulator of mTORC1, as an upstream regulator of differentially expressed genes (DEGs) in βOGT-KO islets (Supplemental Figure 2A). DEGs of these studies were previously reported by Lockridge et al. and Mohan et al. (7, 11). Here we observed an increase in *Tsc2* mRNA transcript as well as protein levels in isolated islets from βOGT-KO mice (Figure 2, A–C). As expected, increased TSC2 protein levels reduce mTORC1 activity in βOGT-KO islets (Figure 2, B and D). If OGT regulates mTORC1 positively via O-GlcNAc, then β cells with OGA deletion (hyper–O-GlcNAcylation) should show increased mTORC1 activity. Indeed, islets from the βOGA-KO show decreased *Tsc2* mRNA or protein levels (Figure 2, E–G) and increased levels of phosphorylated S6 at Ser240, indicating increased mTORC1 activity (Figure 2, F and H). We tested this relationship in islets from patients who are lean, and treatment with Thiamet-G (TMG), an OGA inhibitor, led to increased intensity of RL2 and mTORC1 activity (Figure 2I). These data suggest that the OGT/mTOR relationship is intact in both murine and human islets. Next, we investigated whether mTORC1 may reciprocally modulate O-GlcNAcylation. However, increasing mTORC1 signaling did not alter O-GlcNAcylation levels (Supplemental Figure 2B). Conversely, partial Raptor (a critical component of mTORC1) deletion did not change O-GlcNAcylation in β cells (Supplemental Figure 2C), suggesting that modulation of mTORC1 levels does not alter O-GlcNAcylation levels, and thus, OGT may act upstream of mTORC1 in β cells.

### OGT negatively regulates autophagy in β cells.

**Figure 3:**
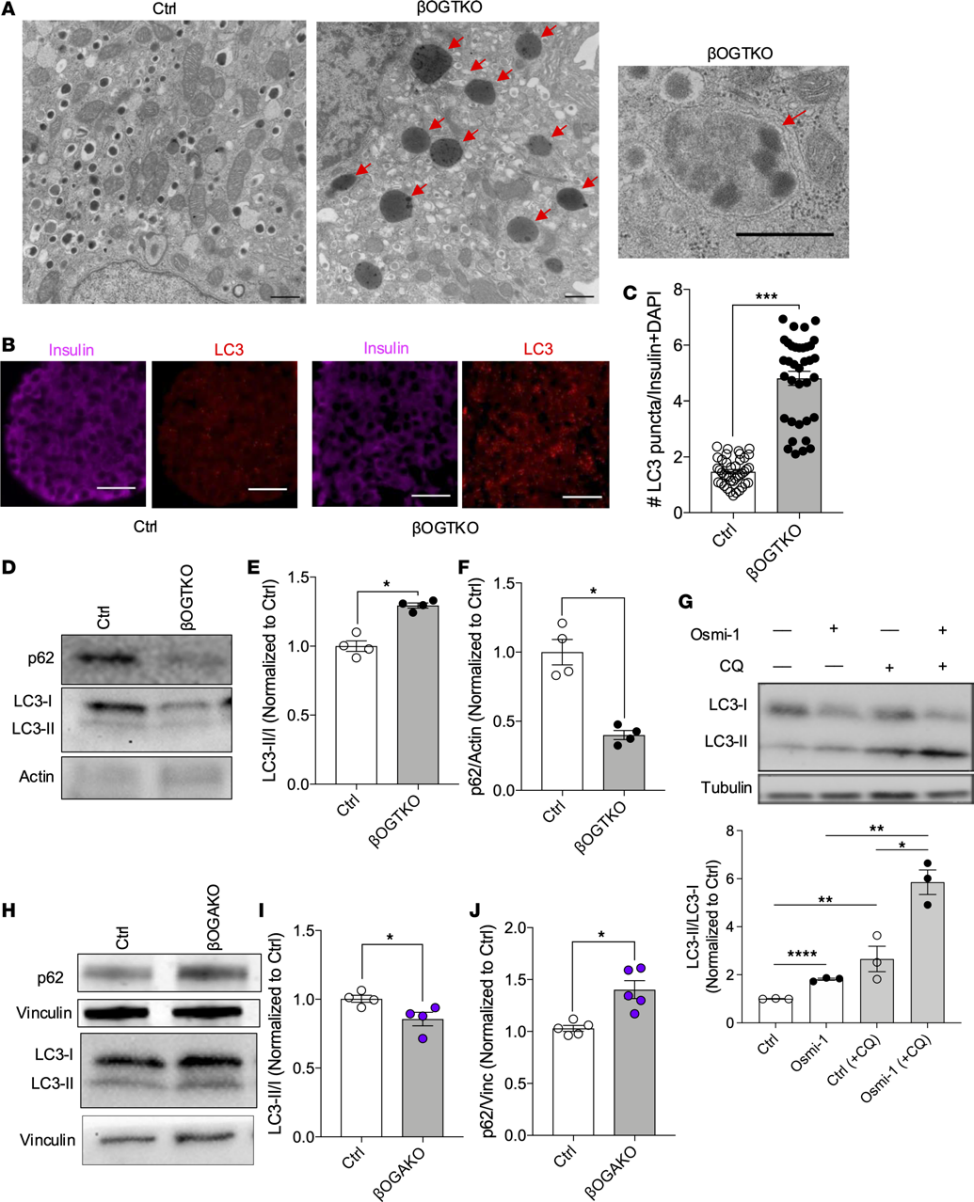
O-GlcNAcylation regulates autophagy in pancreatic β cells. (**A**) Transmission electron microscopy (TEM) image of control and βOGT-KO β cells. Red arrows point to phagophore like structures. Magnification, ×25,000 (small image, right) and ×10,000 (large images, left). Scale bar: 600 nm. (**B** and **C**) Representative immunofluorescence imaging of insulin, LC3 from control, and βOGT-KO pancreas. Magnification, ×400. Scale bar: 25 μm. LC3 puncta quantitation in insulin^+^ cell populations (*n* = 3 mice). (**D**–**F**) Representative immunoblot and analysis of LC3-II, LC3-I, and p62 from control and βOGT-KO islets (*n* = 4–5). (**G**) Representative immunoblot and analysis of LC3-II and LC3-I from INS-1 cells treated with vehicle or OSMI-1 (OGT inhibitor; 50 μM) for 24 hours. Cells treated with chloroquine (CQ; 10 μM) were treated 2 hours prior to collection (*n* = 3). (**H**–**J**) Representative immunoblot and analysis of LC3-II, LC3-I, and p62 from control and βOGA-KO islets (*n* = 4–5). Statistical analysis by Student’s *t* test (**C**, **E**, **F**, **I**, and **J**) or 1-way ANOVA (**G**). *P ≤ 0.05, **P < 0.01, ***P < 0.001, ****P < 0.0001.

mTORC1 acts on various signaling nodes to modulate β cell growth and function, including inhibition of autophagy. However, the role of OGT in autophagy has not been explored in β cells. Analysis of ultraresolution structure by transmission electron microscopy (TEM) on βOGT-KO β cells revealed an increased number of phagophore-like, double-membraned structures (Figure 3A). We tested whether the autophagic process is dysregulated in an O-GlcNAc–dependent manner. Immunofluorescence imaging of OGT deficient β cells revealed increased LC3 puncta (Figure 3, B and C). We corroborated these data with an immunoblot of control and βOGT-KO islets, showing increased lipidation of LC3, indicative of autophagosome formation. A decrease in p62 expression also indicated increased protein turnover via autophagy (Figure 3, D–F). Using a pharmacological inhibitor of OGT, OSMI-1, we detected increased LC3 lipidation, which is further augmented in the presence of chloroquine, an inhibitor of autophagy flux (Figure 3G). These data suggest chloroquine can still block autophagy flux in cells with reduced OSMI-1. Therefore, with reduced OGT, autophagic flux was increased.

To strengthen the evidence that inhibiting O-GlcNAcylation promotes autophagy, we assessed LC3-II levels under conditions of increased O-GlcNAcylation. In βOGA-KO islets, where O-GlcNAcylation was elevated, we observed reduced LC3 lipidation and increased p62 expression (Figure 3, H–J), indicating that autophagy was inhibited in these islets. Overall, our data suggest that O-GlcNAcylation negatively regulates autophagy in β cells, as OGT deletion increased autophagy while OGA deletion decreased it.

### mTORC1 restoration ameliorates hyperglycemia and glucose intolerance in βOGT-KO mice.

**Figure 4:**
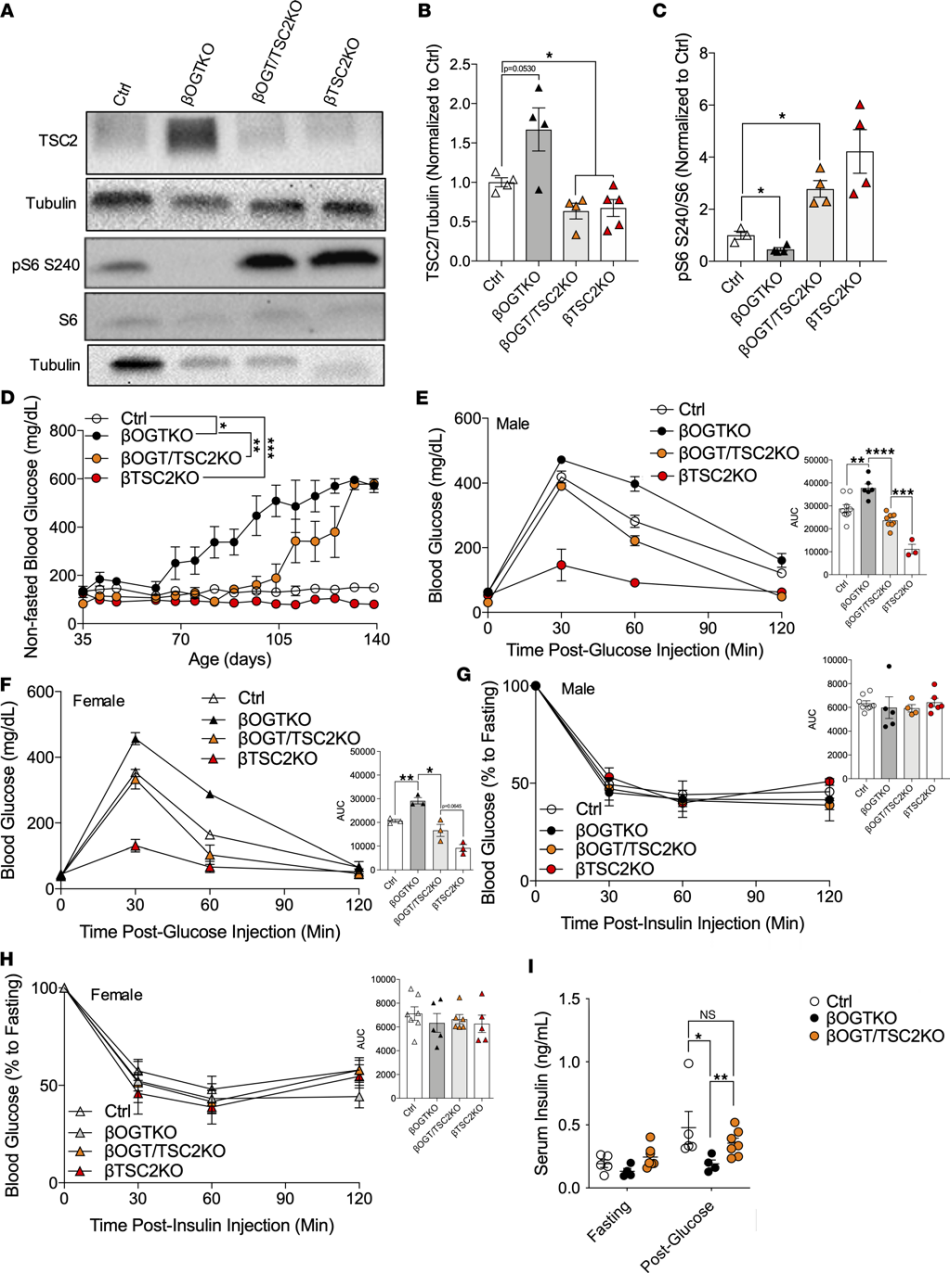
mTORC1 restoration ameliorates β cell dysfunction in βOGT-KO mice. (**A**–**C**) Representative immunoblot and analysis of TSC2, pS6 S240, and S6 from control, βOGT-KO, βOGT/TSC2-KO, and βTSC2-KO islets (*n* = 4–5). (**D**–**H**) Nonfasted blood glucose over time, i.p. glucose tolerance (glucose 2 g/kg i.p.), and insulin tolerance test (insulin 0.75U/kg i.p.) from male and female control, βOGT-KO, βOGT/TSC2-KO, and βTSC2-KO mice (*n* = 3–8). (**I**) In vivo GSIS assay from male control, βOGT-KO, and βOGT/TSC2-KO mice (*n* = 4–7). *P ≤ 0.05, Ctrl versus βOGT-KO or βOGT/TSC2-KO, **P < 0.01, ***P < 0.001, ****P < 0.0001. Statistical analysis by 1-way (**B** and **C**, AUC for **E**–**H**) and 2-way ANOVA (**D** and **I**).

We tested whether restoration of upstream mTORC1 signaling is sufficient to rescue the diabetes observed in the βOGT-KO mice. We deleted TSC2 in the background of βOGT-KO (βOGT/TSC2-KO), and βTSC2-KO was also used as a control in all the in vivo studies. We show efficient deletion of TSC2 and restoration of mTORC1 activity in the βOGT/TSC2-KO islets (Figure 4, A–C). By rescuing mTORC1 activity in βOGT-KO mice, we delayed the development of hyperglycemia in these mice (Figure 4D). While we observed a hyperglycemia-associated decrease in body weight in βOGT-KO after 80 days of age, we observed normalization of body weight in the βOGT/TSC2-KO (Supplemental Figure 3A). Next, we tested their glucose tolerance, insulin tolerance, and glucose-stimulated insulin secretion (GSIS) prior to when the βOGT-KO mice developed hyperglycemia and reduced body weight. At 7–8 weeks of age, we show that βOGT/TSC2-KO mice had improved glucose tolerance in both males and females (Figure 4, E and F). We observed normal insulin tolerance between βOGT-KO, βOGT/TSC2-KO, and respective controls (Figure 4, G and H). An in vivo GSIS assay revealed full rescue in insulin secretion in βOGT/TSC2-KO (Figure 4I and Supplemental Figure 3B), prompting us to assess β cell mass and islet insulin secretion in these models.

### mTORC1 preferentially affects β cell mass, but not insulin biosynthesis, without OGT.

**Figure 5:**
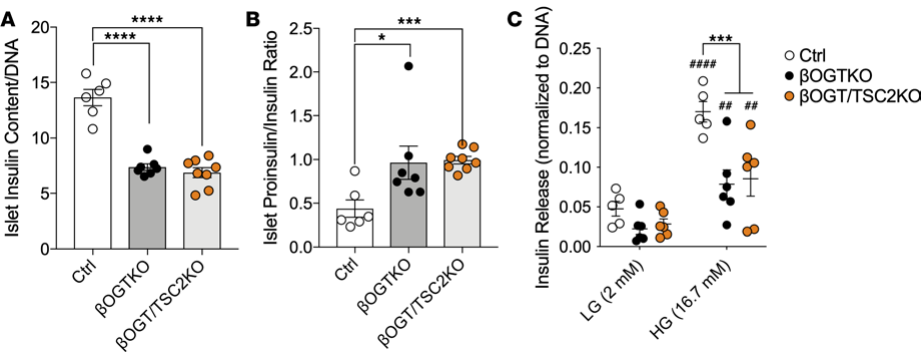
mTORC1 is insufficient modulate insulin biosynthesis in the absence of O-GlcNAcylation. (**A**–**C**) Islet insulin content, proinsulin-to-insulin content ratio, and in vitro islet GSIS assay from control, βOGT-KO, and βOGT/TSC2-KO islets (*n* = 5–8). Statistical analysis by 1-way (**A** and **B**) and 2-way ANOVA (**C**). *P < 0.05, ***P < 0.001, ****P < 0.0001, control or βOGT-KO or βOGT/TSC2-KO. ^##^*P* ≤ 0.01, ^###^*P* < 0.001, LG vs HG.

**Figure 6:**
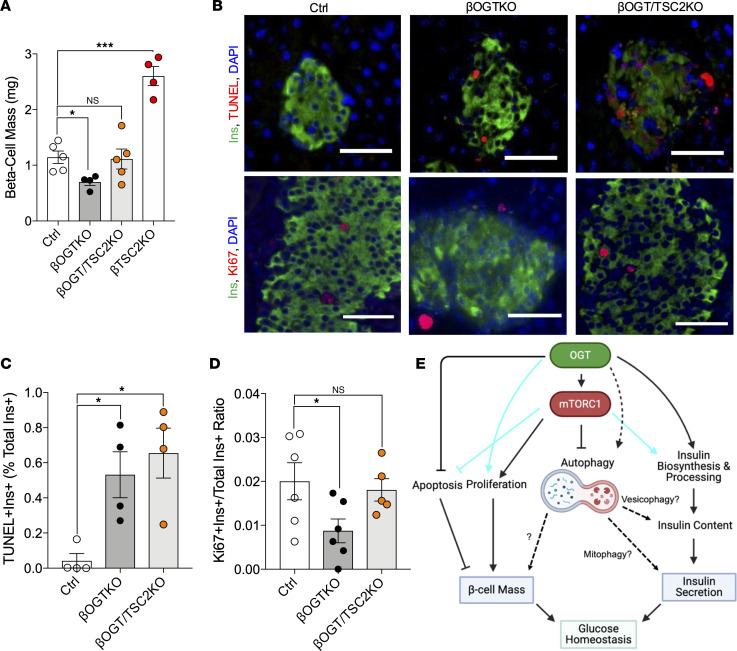
Pancreatic β cell proliferation is primarily driven by mTORC1, independently of O-GlcNAcylation. (**A**–**D**) Ex vivo β cell mass and immunofluorescence-based analysis of apoptosis (TUNEL) and proliferation (Ki67) of insulin^+^ β cells from control, βOGT-KO, and βOGT/TSC2-KO pancreas (*n* = 4–6). Scale bar: 25 um. Statistical analysis by 1-way ANOVA. Total original magnification, ×200. *P ≤ 0.05, ***P < 0.001. (**E**) Proposed model. OGT positively and negatively modulates mTORC1 and autophagy, respectively, in pancreatic β cells. Mechanistically, OGT regulate apoptosis and proliferation to maintain proper β cell mass, and it modulates insulin biosynthesis and processing to control insulin content and secretion. While mTORC1 can gate apoptosis and positively signal insulin biosynthesis and processing, without OGT, it is unable to do so, suggesting that OGT is a dominant signal for these pathways in β cells (light blue line). Conversely, while OGT has been shown to modulate cell proliferation, mTORC1 can induce proliferation in the absence of O-GlcNAcylation (light blue line). Future direction includes studying the downstream effect of altered autophagy (e.g., vesicophagy, mitophagy) that may regulate β cell function. Statistical analysis by Student’s *t* test and 1-way ANOVA. Significance *P* ≤ 0.05.

mTORC1 signaling is a critical regulator for both β cell mass and function. To assess the contributing factors to glucose tolerance and in vivo insulin secretion observed in βOGT/TSC2-KO mice, we assessed islet insulin secretion and β cell mass ex vivo. First, we found that islet insulin content deficit is not rescued in βOGT/TSC2-KO, compared with βOGT-KO islets (Figure 5A and Supplemental Figure 4A), suggesting defects in insulin biosynthesis. Also, proinsulin-to-insulin ratio and carboxypeptidase E (CPE) expression are significantly increased and reduced, respectively, in βOGT/TSC2-KO islets compared with βOGT-KO islets (Figure 5B and Supplemental Figure 4, B and C), suggesting that the insulin processing in OGT-deficient β cells is not rescued by increasing mTORC1 signaling. In addition to insulin content, glucose-stimulus coupling mechanisms such as glucose metabolism and ATP synthesis from the mitochondria are important factors promoting insulin secretion, and mTORC1 plays an important role in mitochondrial biogenesis and function. Despite rescuing mTORC1 signaling, we observed defects in mitochondrial response to glucose and ATP-linked respiration in both βOGT/TSC2-KO and βOGT-KO islets (Supplemental Figure 4, D–I). Additionally, the extracellular acidification rate, a proxy for glycolysis, was reduced in βOGT/TSC2-KO islets (Supplemental Figure 4, J and K). This was consistent with defects in islet GSIS, still observed in βOGT/TSC2-KO islets (Figure 5C). Next, we assessed β cell mass in βOGT/TSC2-KO mice. Though no differences in pancreas mass were detected among genotypes (Supplemental Figure 5A), we observed normalized β cell mass in βOGT/TSC2-KO mice (Figure 6A and Supplemental Figure 5B), suggesting that improved glucose tolerance is largely due to rescue in the β cell abundance. mTORC1 signaling regulates biomass through modulation of cell proliferation and apoptosis. Mechanistically, while we observed no rescue in β cell apoptosis (TUNEL), we observed an increase in β cell proliferation via Ki67 staining in βOGT/TSC2-KO mice (Figure 6, B–D), suggesting that mTORC1 can drive β cell proliferation and set β cell mass in the absence of O-GlcNAcylation.

### Overlapping and distinct pathways regulated by mTORC1 and OGT.

As key nutrient-sensing proteins, OGT and mTORC1 regulate multiple signaling nodes to orchestrate β cell cellular function and health. To investigate the differential signaling between these 2 major pathways, we performed a phospho-protein antibody array to assess and understand changes in the phosphorylation signaling cascade in the islets of βOGT-KO, βTSC2-KO, and βOGT/TSC2-KO. This approach helps delineate the signaling relationship between OGT and mTORC1, particularly since some mTORC1 targets are themselves kinases. Using a thresholding fold change of 20% to control, we identified 67, 121, and 103 differential phospho-proteins in βOGT-KO, βOGT/TSC2-KO, and βTSC2-KO, respectively. KEGG (https://www.genome.jp/kegg/) and GO-term (https://geneontology.org/) analyses of these differential phospho-protein expressions showed converging pathways in cell cycle, apoptosis, MAPK, and Akt signaling pathways (Supplemental Figure 6A). To identify common or differentially regulated signaling, we overlapped these changes between βOGT-KO, βOGT/TSC2-KO, and βTSC2-KO (Supplemental Figure 6B). The 12 overlaps with the same directionality of change between βOGT-KO and βOGT/TSC2-KO represent phosphorylation changes that are OGT dependent. Seventeen of 26 overlaps with the same directionality between βOGT/TSC2-KO and βTSC2-KO are identified as phosphorylation changes that are mTORC1 dependent. We focused on the 9 phospho-proteins that are commonly altered in all 3 genotypes, and of the 9, we identified p-MKK4 (S80), p-calmodulin (T79), and p-4E-BP (T45) as most substantially changed (Supplemental Figure 6C). Consistent with the proposed model, phosphorylation of 4EBP, a known downstream target of mTORC1, is reduced in βOGT-KO islets but is increased in βOGT/TSC2-KO and βTSC2-KO islets. p-MKK4 S80 is reduced in both βOGT-KO and βOGT/TSC2-KO and increased in βTSC2-KO islets, suggesting that while this pathway can be mTORC1 regulated, it is critically dependent on OGT signaling. Calmodulin phosphorylation at T79 is increased in βOGT-KO islets with relative reduction observed in βOGT/TSC2-KO islets, suggesting a potential intersection of OGT and mTORC1 in this pathway. These data show that OGT and mTORC1 have both overlapping and distinct pathways in regulating molecular signaling cascades to orchestrate β cell function.

## Discussion

T2D develops when β cells fail to respond to nutrients and produce enough insulin to tightly regulate blood glucose levels. T2D risk is linked to obesity, as excess nutrients associated with obesity can exacerbate β cell dysfunction at multiple levels, including autophagy, mitophagy, ER stress, and apoptosis. The mechanism through which nutrient excess amplifies β cell health and function remains elusive. While it is generally acknowledged that nutrient signaling through mTORC1 and OGT are important in islet fitness and function, how they crosstalk is largely unknown in β cells. O-GlcNAcylation and mTORC1 are key regulators of cell signaling and are involved in various physiological and pathological processes. Their interaction to enhance cell function and survival is expected, especially in metabolically active cells like β cells, which are responsible for sensing nutrients to regulate glucose homeostasis. In this study, we tested that O-GlcNAcylation, a nutrient-sensitive process, positively regulates mTORC1 activity and its downstream effector to control β cell mass and function (Figure 6E). Utilizing both loss- and gain-of-function models of O-GlcNAcylation, we show that the balance of this posttranslational modification was critical for β cell function and glucose homeostasis.

While a few studies have described the crosstalk between O-GlcNAcylation and mTOR in the context of metabolic dysregulation, such as in cancer (12), their interaction under normal metabolic condition in β cells remains unexplored. In this study, we demonstrate that mTORC1 signaling was regulated by O-GlcNAcylation, with reduced O-GlcNAcylation leading to decreased mTORC1 activity and with increased O-GlcNAcylation enhancing mTORC1 activity in β cells. In different cell types, manipulation of O-GlcNAcylation has shown varying effects on mTORC1 activities (12–14). In neurons and HEK293T cells, OGT positively regulate mTOR signaling (13, 15). However, in mouse embryonic stem cells (mESCs), OGT deletion led to mTORC1 hyperactivation (16). In HEK293T cells, O-GlcNAcylation of Raptor at threonine 700 facilitates the interactions between Raptor and Rag GTPases and promotes the translocation of mTOR to the lysosomal surface, consequently activating mTORC1 (15). OGT modulates energy homeostasis in the body in a cell type–specific manner (17–19), and this is a possible explanation why OGT-mTORC1 relations are context or tissue dependent. We postulate that OGT’s lack of strict consensus sequence (20) may contribute to its varying targets in different tissue types. At the molecular level, OGT may regulate mTORC1 activity through direct O-GlcNAcylation of its components such as Raptor (15); however, based on the results of the βOGT/TSC2-KO islets, where mTORC1 activity is fully rescued in the absence of O-GlcNAcylation, O-GlcNAcylation of the mTORC1 component is not a prerequisite to its activity. We also assessed whether mTORC1 can modulate O-GlcNAcylation levels in β cells. Our data suggest that increased mTORC1 activity, via deletion of TSC2, does not increase O-GlcNAcylation in β cells. In breast cancer cells, elevated mTOR/c-MYC was previously shown to increase the expression of OGT and O-GlcNAcylation (21). In pancreatic β cells, our data suggest that OGT is upstream of mTORC1.

In this study, we also show that autophagy was normally regulated negatively by O-GlcNAcylation, where deletion of OGT or OGA led to increased or decreased autophagy, respectively, in β cells. Several studies support our findings in non–β cells. For example, in the brain of the mouse, deletion of OGT in cortical astrocytes increases autophagy (22). OGT overexpression reduces autophagy, whereas OGT reduction via RNAi increases basal autophagy in *Drosophila melanogaster* (23). It is important to recognized, however, that deletion of OGT in liver or heart reduces autophagy in fasting states (18, 24). mTOR is a known regulator of autophagy. It is possible that downregulation of mTOR signaling in OGT-deficient cells could activate autophagy. In neurons, inhibition of OGT led to increased mTOR-dependent autophagy via stimulation of autophagic flux (13). Our data also support that stimulation of autophagic flux occurs when OGT is blocked in β cells. At the molecular level, using in vitro studies done in the heart show that O-GlcNAcylation of key proteins such as ULK1/2 (unc-51 like autophagy activating kinase, downstream of mTORC1 signal) is important for the initiation of autophagy and phagophore formation at the ER (25). Thus, O-GlcNAcylation may directly control autophagy through several autophagy proteins such as ULK1/2 as shown in non–β cells (26–28) or indirectly through mTORC1 phosphorylation on ULK1/2 (29). Additionally, O-GlcNAcylation can indirectly affect TFEB localization, a master transcriptional factor for lysosomal biogenesis, through Raptor (15), which may contribute to increased phagophore-like structures seen in our TEM imaging of βOGT-KO β cells. Further investigation focused on O-GlcNAc modifications of specific autophagy proteins and the role of mitophagy and lysosomal function and neogenesis in OGT/OGA-deficient β cell models are warranted to understand the dynamic role of the O-GlcNAc nutrient-sensor pathway in regulating mitochondrial health and turnover and to understand regulatory input of OGT in autophagy in relation to β cell function and glucose homeostasis.

The mTORC1 signaling cascade is critical for both β cell mass and function. mTORC1 signals to increase proliferation and inhibit apoptosis, leading to increased β cell mass and increased in vivo insulin secretion (30–32). Indeed, our data support that the β cell mass deficit in OGT-deficient mice is largely driven by loss of mTORC1, as concomitant deletion of TSC2 — thereby increasing mTORC1 — leads to normalization of β cell mass. Upregulation of Akt, upstream of mTORC1, also rescues β cell mass deficit in OGT-KO (5). While OGT targets the proteins of translational machinery and cell cycle (33, 34), our data suggest that mTORC1 signaling may play a more dominant role in regulating cellular proliferation than OGT in the context of maintaining β cell mass. In addition to its regulation of β cell mass, mTORC1 can modulate cellular processes such as insulin biosynthesis/processing and glucose-stimulus coupling (35). mTORC1 affects overall insulin content levels, in part, through insulin transcription via Pdx1 (36) and processing through CPE expression (35). Pdx1 is a target of OGT in β cells (37, 38), and CPE expression is shown to be regulated through an OGT-dependent eIF4G1 translational mechanism (6). Enhancing mTORC1 through deletion of TSC2 was not sufficient to rescue the effect of OGT on insulin and proinsulin content and CPE protein levels. These data suggest that, while mTORC1 can affect the insulin biosynthesis pathway, direct modulation of specific target proteins by OGT (downstream of mTORC1 such as EIF4G1) is required and critical for the maintenance of islet insulin content. Additionally, mTORC1 can regulate insulin secretion through regulation of mitochondrial biogenesis and function (39, 40). β Cell TSC2 deletion shows increased GSIS in part due to enhanced mitochondrial biogenesis (41). However, in our model of βOGT/TSC2-KO, the mTORC1 restoration failed to rescue the mitochondrial dysfunction observed in βOGT-KO islets, suggesting that OGT may play a more central role in regulating mitochondrial function. Indeed, OGT has a mitochondrial isoform and targets specific mitochondrial proteins, such as leucine-rich PPR-containing protein and mitochondrial aconitate hydratase to regulate mitochondrial biogenesis and metabolism (42, 43). βOGT-KO islets exhibit abnormal mitochondrial morphology and dysfunction (7). Overall, our data suggest that, for insulin secretion pathway, O-GlcNAcylation of specific target proteins may be required to modulate β cell insulin production and release, regardless of mTORC1 signaling.

In an attempt to study the downstream phosphorylation crosstalk between OGT and mTORC1, we performed a phospho-protein antibody array on islets from control, βOGT-KO, βOGT/TSC2-KO, and βTSC2-KO mice. Here we report that phospho-signaling in MAPK MKK4 and calmodulin may be altered in O-GlcNAc–dependent and O-GlcNAc/mTORC1–dependent manner, respectively. The consistent decreases in inhibitory phosphorylation of MKK4 (44, 45) in βOGT-KO and βOGT/TSC2-KO, as well as identifying the MAPK cascade in the GO and KEGG analyses, suggest that, in the absence of O-GlcNAcylation, MAPK and its downstream signaling JNK and NF-κB (46) may be increased. Reports have suggested that elevation in these signaling pathways inhibits insulin biosynthesis (47, 48), which is congruent with the defect in insulin biosynthesis observed in both βOGT-KO and βOGT/TSC2-KO islets. It is likely that O-GlcNAcylation of the MKK4 pathway may be an important signal for maintaining insulin biosynthesis. Future studies will focus on delineating the effect of O-GlcNAcylation on specific target proteins of this pathway and how they interact with insulin synthesis.

Some of the limitations of the study, may include using the constitutive insulin promoter–driven Cre recombinase that could result in some compensatory or adaptive changes starting from early life that might affect signaling pathways. Previous studies have shown that different RIP-Cre mouse lines have widespread Cre activity in the brain, including the hypothalamus (49). In this study, we used the RIP-Cre*^Herrera^* (50), where we previously reported that OGT expression in the hypothalamus of βOGT-KO mice is not altered (5), and we have established no Cre-effect allowing us to use littermates as control. Additionally, the specific molecular mechanisms underlying the complex connection between OGT, mTORC1, and autophagy in β cells warrants further investigation. Ongoing work in the lab focuses on identifying specific targets of OGT in β cells and how O-GlcNAcylation affect their function.

In conclusion, our study highlights the balancing act of the nutrient sensor OGT and mTORC1 in regulating pancreatic β cell mass and function. The interplay between these nutrient-sensing pathways is complex but presents a promising area for future research and therapies to improve β cell health and combat diabetes mellitus. By gaining a deeper understanding of the molecular basis of β cell dysfunction, we may ultimately contribute to the development of more effective treatments for this prevalent metabolic disorder.

## Methods

### Sex as biological variable.

Both male and female animals were used for base in vivo characterization of the glucose tolerance phenotype. Tissues from male animals were used for ex vivo and in vitro phenotyping, as our previous work in OGT-deficient mouse model revealed no sexual dimorphism. We expect our findings to be relevant for both males and females.

### Human donor islets.

Human donor islets were procured through Integrated Islet Distribution Program (IIDP) and University of Alberta (Supplemental Table 1), from individuals who are lean (BMI < 25) or have obesity (BMI > 30); all without diabetes and A1C levels less than 6%. Live islets were rested overnight (16–18 hours) in a 37°C, 5% CO_2_ incubator prior to collection or further testing. For nutrient stimuli, islets were incubated in Krebs (114.5 NaCl, 4.7 KCl, 1.2 KH_2_PO_4_, 1.1 MgSO_4_-7H_2_O, 8 HEPES, 1 CaCl_2_-2H_2_O, 10 NaHCO_3_ [in mM] and 0.08% w/v BSA; Sigma-Aldrich) with combinations of 2 mM glucose (L), 16.7 mM glucose (H), amino acids (A) (2,000 glutamine, 100 isoleucine, 200 leucine, 230 valine, 50 arginine, 30 methionine, 90 alanine [μM]), or 100 μM palmitate (P) (precomplexed 6:1 to BSA) for 6 hours and frozen down for immunoblot analysis. Different sets of islets were treated with TMG, an OGA inhibitor (MilliporeSigma; 30 μM), for 12 hours and collected for histogel embedment.

### Animal models and in vivo mouse procedures.

To generate pancreatic β cell–specific deletion of target genes, mice with 1 allele of RIP-Cre recombinase (rat insulin promoter Cre recombinase; gifted by Pedro Herrera, University of Geneva, Geneva, Switzerland) were crossed with the following loxP-flanked genes in various combinations: OGT^fl/fl^, TSC2^fl/fl^ (purchased from The Jackson Laboratory), Raptor^fl/fl^ (in house), and OGA^fl/fl^ (gifted by John A. Hanover, NIH, Bethesda, Maryland, USA). Whole-body eIF4EBP2 knockout (in house) were used in maternal low protein diet study. Control or eIF4EBP2-KO mice were generated from dams fed either control or LPD (9%, D02041002, Research Diets Inc.) throughout pregnancy. All mice were group-housed under a 14:10 light-dark cycle with ad libitum access to standard chow.

Glucose tolerance tests (GTT) and insulin tolerance tests (ITT) were conducted in overnight-fasted or 6-hour–fasted animals, respectively. Glucose levels were measure immediately preceding and up to 2 hours after an i.p. injection of 2 g/kg glucose (Hospira, Pfizer) or 0.75 U/kg insulin (Humalog, Eli Lilly). For in vivo GSIS, plasma samples were assayed from facial vein blood collected from overnight-fasted mice before and 5 minutes after a 3 g/kg injection of glucose.

### Cell culture, islet isolation, and in vitro testing.

INS-1 cells (gifted by Peter Arvan, University of Michigan, Ann Arbor, Michigan, USA) were maintained in RPMI 1640 (Corning), supplemented with 10% FBS and penicillin/streptomycin (GenClone, Thermo Fisher Scientific), in a humidified 37°C, 5% CO_2_ incubator. OSMI-1 (MilliporeSigma) and chloroquine (MilliporeSigma) were dissolved in DMSO and water, prior to use, respectively. For autophagy assessment, INS-1 cells were treated with DMSO or OSMI-1 (50 μM) for 24 hours, followed by chloroquine (10 μM) for 2 hours, prior to collection.

For primary islet isolation, the pancreas was perfused with 0.75–1 mg/mL of ice-cold collagenase (MilliporeSigma) in HBSS (Thermo Fisher Scientific) through the common bile duct. The inflated pancreas was then carefully dissected and trimmed of fat, followed by digestion for approximately 8–10 minutes at 37°C with manual agitation. The resulting tissue pellet was washed with HBSS containing 2% FBS (GenClone), strained to remove undigested debris, and filtered through a 70 μm cell strainer (Thermo Fisher Scientific). After washing the retained material off the filter screen, islets were manually picked and transferred into warm islet media (RPMI supplemented with l-glutamine [Corning], 10% FBS, 100 IU/mL of penicillin, 100 g/mL of streptomycin, and 5 mM glucose). The isolated islets were then cultured in complete media overnight in a humidified 37°C, 5% CO_2_ incubator before further testing or collection.

For in vitro islet GSIS, islets were incubated for 2 hours in sterile Krebs containing (in mM): 114.6 NaCl, 4.7 KCl, 1.2 KH_2_PO_4_, 1.1 MgSO_4_-7H_2_O, 8 HEPES, 1 CaCl_2_-2H_2_O, 10 NaHCO_3_, and 0.08% BSA w/v. Subsequently, 8–10 size-matched islets were transferred into 8 μm cell culture inserts and sequentially incubated in low-glucose (LG) and high-glucose (HG) solutions for 30 minutes each. The collected supernatant was analyzed for insulin concentration using ELISA (Alpco). The inserts were then processed for insulin and DNA content assessment. The insulin content of both naive and post-GSIS islets was normalized to DNA.

### Western blot.

Protein lysates (25–40 μg) in RIPA buffer with 0.1% SDS and protease/phosphatase inhibitors were quantified using Pierce BCA protein assay. They were resolved by SDS-PAGE, transferred to a polyvinylidene difluoride (PVDF) membrane, and blocked with 5% nonfat dry milk. Primary antibodies (TSC2 [4308; Cell Signaling Technology], pS6 S240 [5364; Cell Signaling Technology], S6 [sc-74459; Santa Cruz Biotechnology Inc.], RL2 [ab2739; Abcam], OGT [24083; Cell Signaling Technology], OGA [SAB4200267; MilliporeSigma], mTOR [2983; Cell Signaling Technology], Actin [4967; Cell Signaling Technology], Vinculin [E1E9V; Cell Signaling Technology], Tubulin [2146; Cell Signaling Technology], LC3B [3868; Cell Signaling Technology], p62 [5114; Cell Signaling Technology]) were applied, followed by horseradish peroxidase–conjugated secondary antibodies. The blot was visualized using SuperSignal West Pico PLUS chemiluminescence or LiCor IR detection. Densitometry analysis was performed using ImageJ software (NIH). For multiprotein bands in RL2, bands between 50 kDa and 200 kDa were analyzed for quantification. Band sizes with nonspecific signal in 1 or more samples were excluded from analysis from all samples.

### Quantitative PCR mRNA analysis.

For RNA analysis, islets were washed in PBS before being quickly frozen and stored at –80°C until RNA isolation. RNA isolation was performed using the RNA MiniPrep kit following the provided instructions (Qiagen). RNA concentration was measured using a Tecan microplate spectrophotometer. cDNA was synthesized from islet RNA using a high-capacity cDNA reverse transcription kit (Applied Biosystems). Relative gene expression was evaluated on an Applied Sciences 7900HT Real-Time PCR system using SYBR green (Applied Biosciences) and the ΔΔCT method, with normalization to β-actin.

### Ex vivo pancreas β cell mass, immunofluorescence imaging, and TEM.

Formalin-fixed pancreatic tissues were embedded in paraffin and cut into 5 μm sections. After deparaffinization and rehydration, antigen retrieval was performed using citrate buffer. The sections were then incubated overnight at 4°C with primary antibodies against insulin (R00261-2; DAKO), RL2 (ab2739; Abcam), pS6 S240 (5364; Cell Signaling Technology), or LC3B (3868; Cell Signaling Technology). For immunofluorescence imaging, secondary antibodies conjugated to FITC, Cy3, or Cy5 were used, and the sections were cover slipped with DAPI-containing mounting media. Imaging was done using a Keyence microscope with a motorized stage for whole-pancreas scanning.

To assess β cell mass, ImageJ software was used to calculate the ratio of insulin^+^ area to total pancreas area (β cell area). This was then multiplied by the pancreas weight to determine the β cell mass, based on 5 insulin-stained sections taken 200 μm apart through the entire depth of the pancreas. TUNEL staining was performed on pancreas tissues using ApopTag Red In Situ Apoptosis Detection Kit (MilliporeSigma), according to manufacturer instructions. Ki67 antibody (ab15580; Abcam) was used to mark proliferating β cells. Data were analyzed in insulin^+^ cells only.

For TEM, isolated islets fixed with 2.5% glutaraldehyde in 0.1M Sorensen’s Phosphate Buffer (Thermo Fisher Scientific), postfixed in OsO_4_, and stained with 3% uranyl acetate were sectioned using a diamond knife on a Leica Ultracut UCT microtome at a thickness of 70 μm, collected on 200 mesh copper grids. Grids were observed on a Philips CM-12 transmission electron microscope at 60kV.

### Seahorse analysis.

Mouse islets were seeded into wells of XFe96 plates containing 180 μL/well of Seahorse assay medium prepared following the manufacturer’s recommendation. Mitochondrial respiration was measured using the Seahorse XF Cell Mito Stress Test Kit for the Seahorse XFe96 Extracellular Flux Analyzer (Agilent Technologies). Basal respiration was first measured in 2.5 mmol/L glucose media. Islets were then sequentially exposed to 20 mM glucose, 1 μM oligomycin A, 2 μM carbonyl cyanide-p-trifluoromethoxyphenylhydrazone (FCCP), and 0.5 μM antimycin A plus rotenone. OCR and ECAR measurements were normalized to DNA content measured using the Quant-iT PicoGreen dsDNA Assay (Thermo Fisher Scientific).

### Phosphoantibody array.

Primary islets from control, βOGT-KO, βOGT/TSC2-KO, and βTSC2-KO male mice (*n* = 2 samples per genotype) were processed using The Phospho Explorer Antibody Array (Full Moon Biosystems) platform, according to manufacturer’s protocol. The array was analyzed by the manufacturer for signal detection. The phospho-antibody signal was normalized to respective total antibody signal. Datasets from each transgenic islets were analyzed as a fold change to control islets. Signals were rank ordered, and signals with fold-change of greater than 20% were further analyzed in GO, KEGG, and Reactome.

### Statistics.

Data are presented as mean ± SEM and were analyzed using 2-tailed nonparametric, unpaired Student’s *t* test. Multiple outcome data were assessed using repeated measures 2-way ANOVA. For comparisons of 3 or more groups, data were analyzed by 1 way ANOVA with Tukey’s post test. Statistical analyses were performed in GraphPad Prism version 7 with a significance threshold of *P* ≤ 0.05.

### Study approval.

All animal studies were performed in accordance with the University of Minnesota IACUC (no. 2106-39213).

### Data availability.

Data presented in this manuscript, including values for all data points shown in graphs, are available in the Supporting Data Values file or from the corresponding author upon request.

## Author contributions

SJ developed the study. SJ, NE, AN, AW, RM, CK, and EUA designed experiments, generated and analyzed data, and approved the final version. MBR and EBM provided tissue samples and approved the final version. SJ and EUA interpreted the data and wrote and edited the manuscript. EUA conceived the study and was in charge of overall direction of this work. All authors contributed to the article and approved the submitted version.

## Supplementary Material

Supplemental data

Unedited blot and gel images

Supporting data values

## Version 1 10/10/2024

In-Press Preview

## Version 2 12/06/2024

Electronic publication
