# Supplementary material for: Loss of O-GlcNAcylation modulates mTORC1 and autophagy in β cells, driving diabetes 2 progression
Source: JCI Insight. 2024 Dec 6;9(23):e183033. doi: 10.1172/jci.insight.183033 (PMC11623944; doi:10.1172/jci.insight.183033)
Supplement: Supplemental data [file jciinsight-9-183033-s228.pdf]

## Supplemental Figure 1

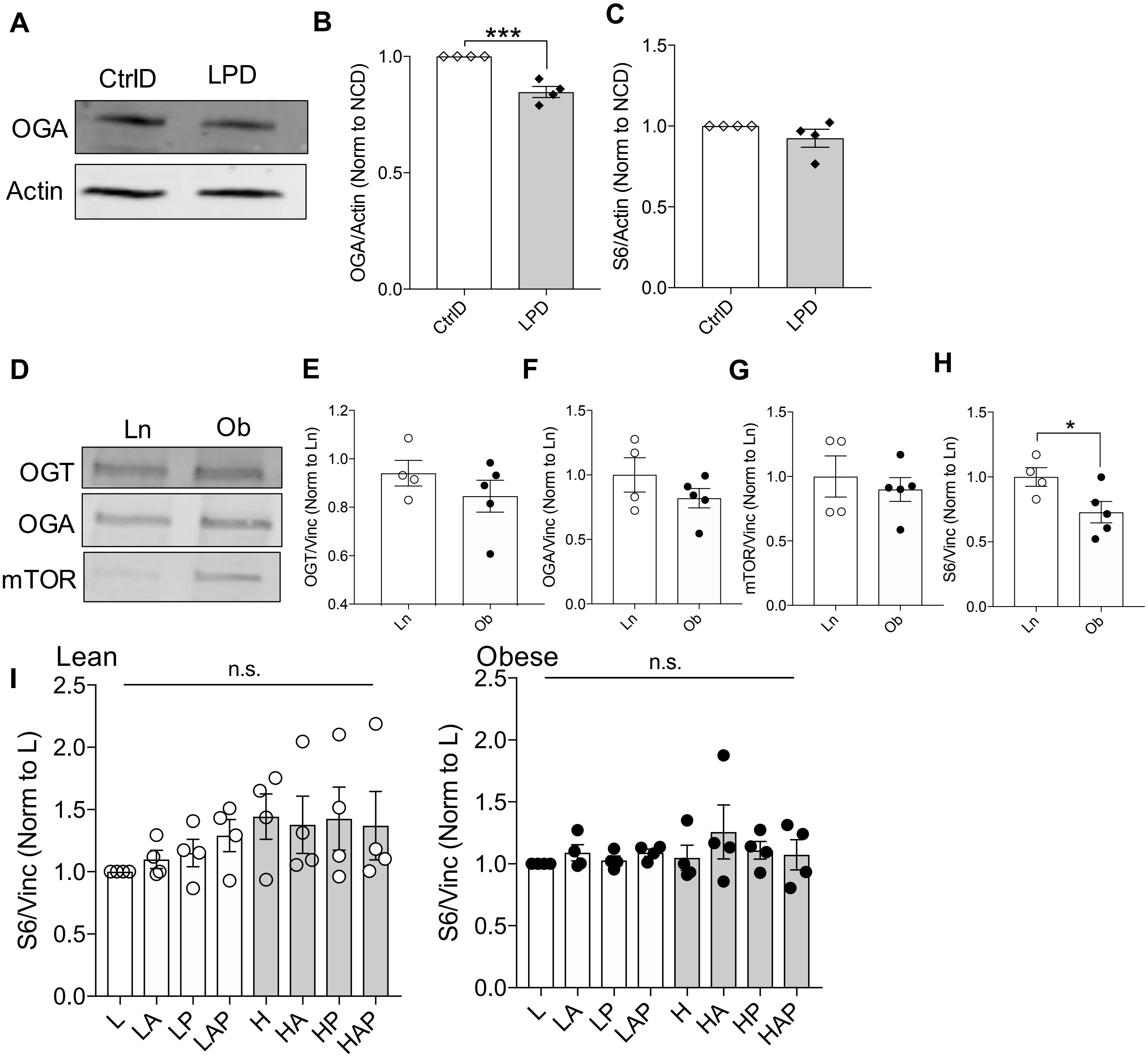

**Supplemental Figure 2**

**A**

| Upstream Regulator | P-value of overlap | OGTKO vs Ctrl |
|---|---|---|
| TSC2 | 9.38E-04 | RNASeq |
| TSC2 | 4.11E-06 | Proteomic |

**B**

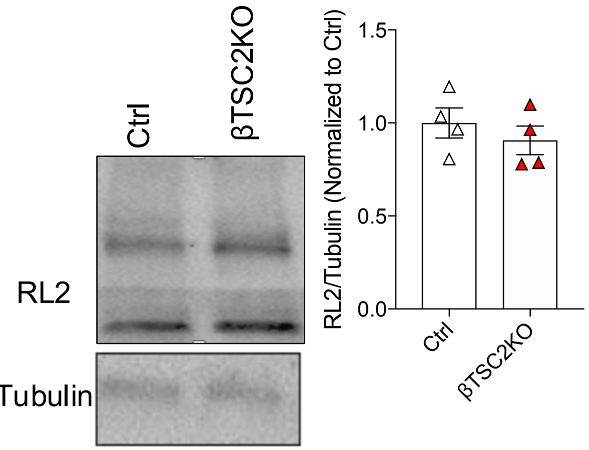


**C**

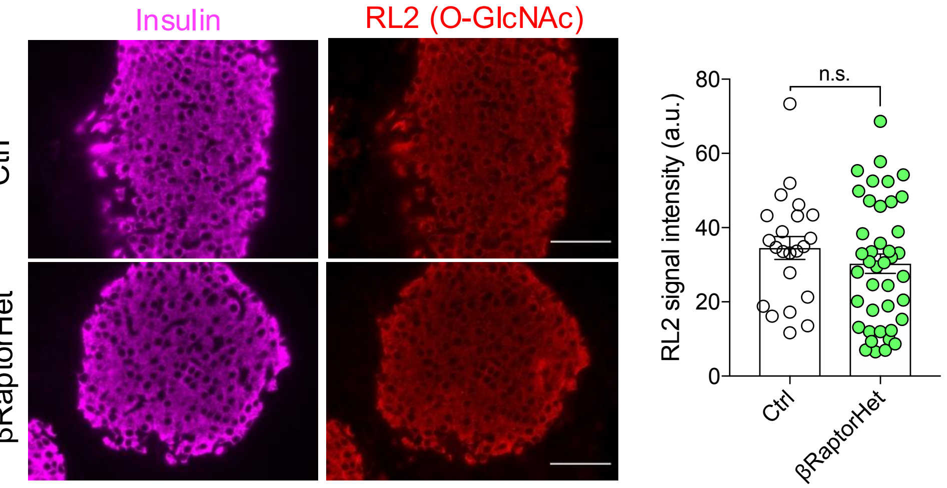

## Supplemental Figure 3

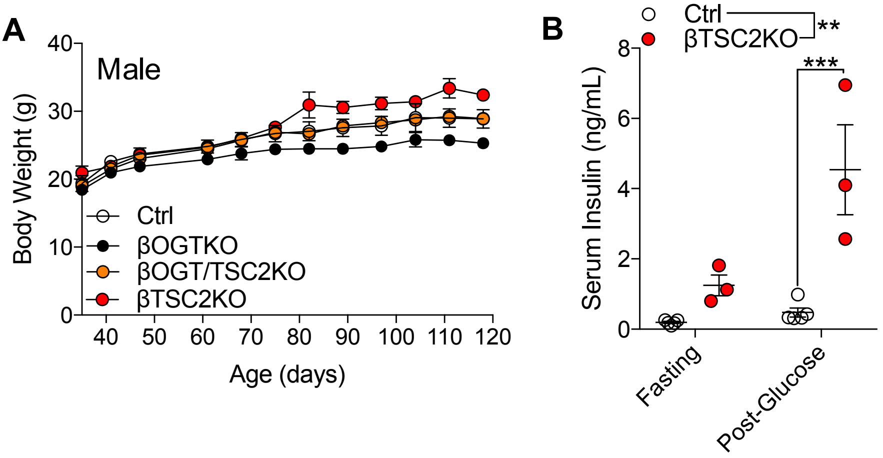

## Supplemental Figure 4

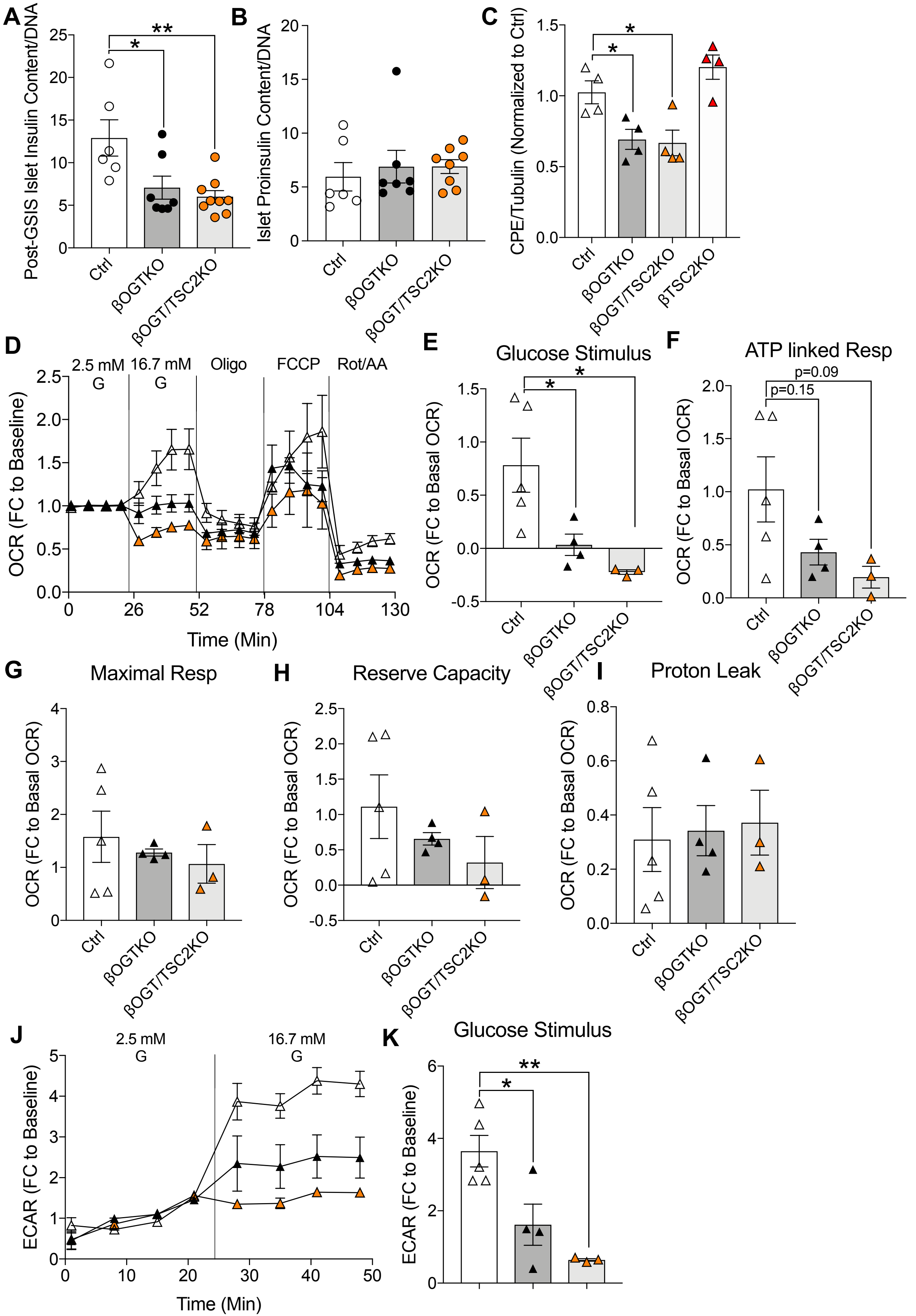

## Supplemental Figure 5

**A**

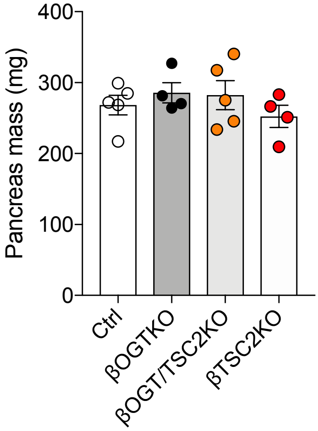


**B**

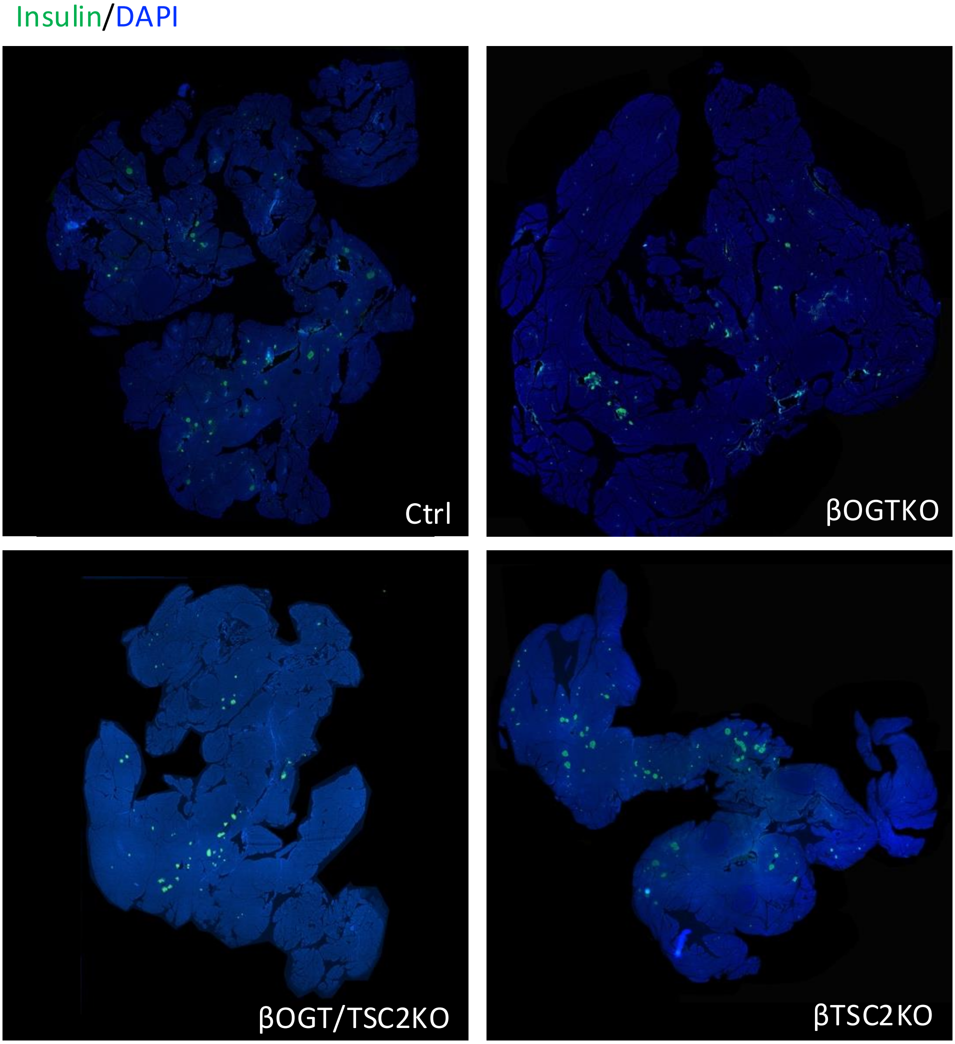

**Supplemental Figure 6**

**A**

| | βOGTKO / Ctrl | βOGT/TSC2KO / Ctrl | βTSC2KO / Ctrl |
|---|---|---|---|
| GO Term<br>KEGG<br>Reactome | Apoptotic process<br>Regulation of Cell Cycle<br>MAPK cascade<br>EGFR tyrosine Kinase<br>Signaling by interleukins | Programmed Cell Death<br>Regulation of Cell Growth<br>MAPK cascade<br>PI3K-AKT signaling<br>Signaling by interleukins | Apoptotic Process<br>MAPK cascade<br>Insulin secretion<br>Interleukin-1 mediated |

**B**

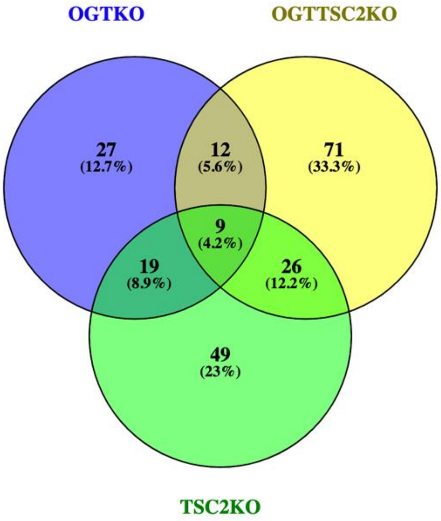


**C**

| | βOGTKO/Ctrl | βOGTTSC2KO/ Ctrl | βTSC2KO/Ctrl |
|---|---|---|---|
| MKK4 (s80) | 0.7889 | 0.7171 | 1.3108 |
| Calmodulin (t79) | 1.8621 | 1.2023 | 0.6972 |
| 4E-BP (t45) | 0.7918 | 1.2897 | 1.3934 |

| Record ID | Gender | Ethnicity/Race | Age (Years) | BMI (kg/m$^2$) | Diabetes Status | Frozen/Live | Source |
|---|---|---|---|---|---|---|---|
| SAMN22997021 | Male | White | 44 | **42** | ND | Live | IIDP |
| SAMN23958504 | Male | White | 42 | **37.9** | ND | Live | IIDP |
| SAMN24579752 | Male | Black/AA | 55 | 24.9 | ND | Live | IIDP |
| SAMN26177826 | Male | White | 52 | 25.1 | ND | Live | IIDP |
| SAMN26527277 | Male | Black/AA | 61 | 22.6 | ND | Live | IIDP |
| SAMN28874962 | Male | White | 32 | 25 | ND | Live | IIDP |
| SAMN30927409 | Male | White | 50 | **36.3** | ND | Live | IIDP |
| SAMN36510137 | Male | White | 63 | **32.1** | ND | Live | IIDP |
| SAMN37871873 | Male | White | 59 | **41.7** | ND | Live | IIDP |
| SAMN37529025 | Male | White | 64 | 23 | ND | Frozen | IIDP |
| SAMN37822573 | Male | White | 54 | 23.1 | ND | Frozen | IIDP |
| SAMN37871873 | Male | White | 59 | **41.7** | ND | Frozen | IIDP |
| SAMN36510137 | Male | White | 63 | **32.1** | ND | Frozen | IIDP |
| SAMN12227196 | Male | White | 51 | **32.8** | ND | Frozen | IIDP |
| R484 | Male | n/a | 33 | 22 | ND | Frozen | U of Alberta |
| R446 | Male | n/a | 64 | **40.7** | ND | Frozen | U of Alberta |
| R472 | Male | n/a | 49 | 20.4 | ND | Frozen | U of Alberta |
| R495 | Male | n/a | 50 | **33.8** | ND | Frozen | U of Alberta |

**Table S1.** Human Islets Donors used in Figure 1. Bold font under "BMI" indicates donors with obesity. ND = non-diabetic. Live islets (SAMN37871873) except were used for Figure 1J-O, S.Figure1I. Frozen islets were used to generate data in Figure 1F-I, S.Figure 1D-H.
