# Supplementary material for: Loss of O-GlcNAcylation modulates mTORC1 and autophagy in β cells, driving diabetes 2 progression
Source: JCI Insight. 2024 Dec 6;9(23):e183033. doi: 10.1172/jci.insight.183033 (PMC11623944; doi:10.1172/jci.insight.183033)

Figure 1

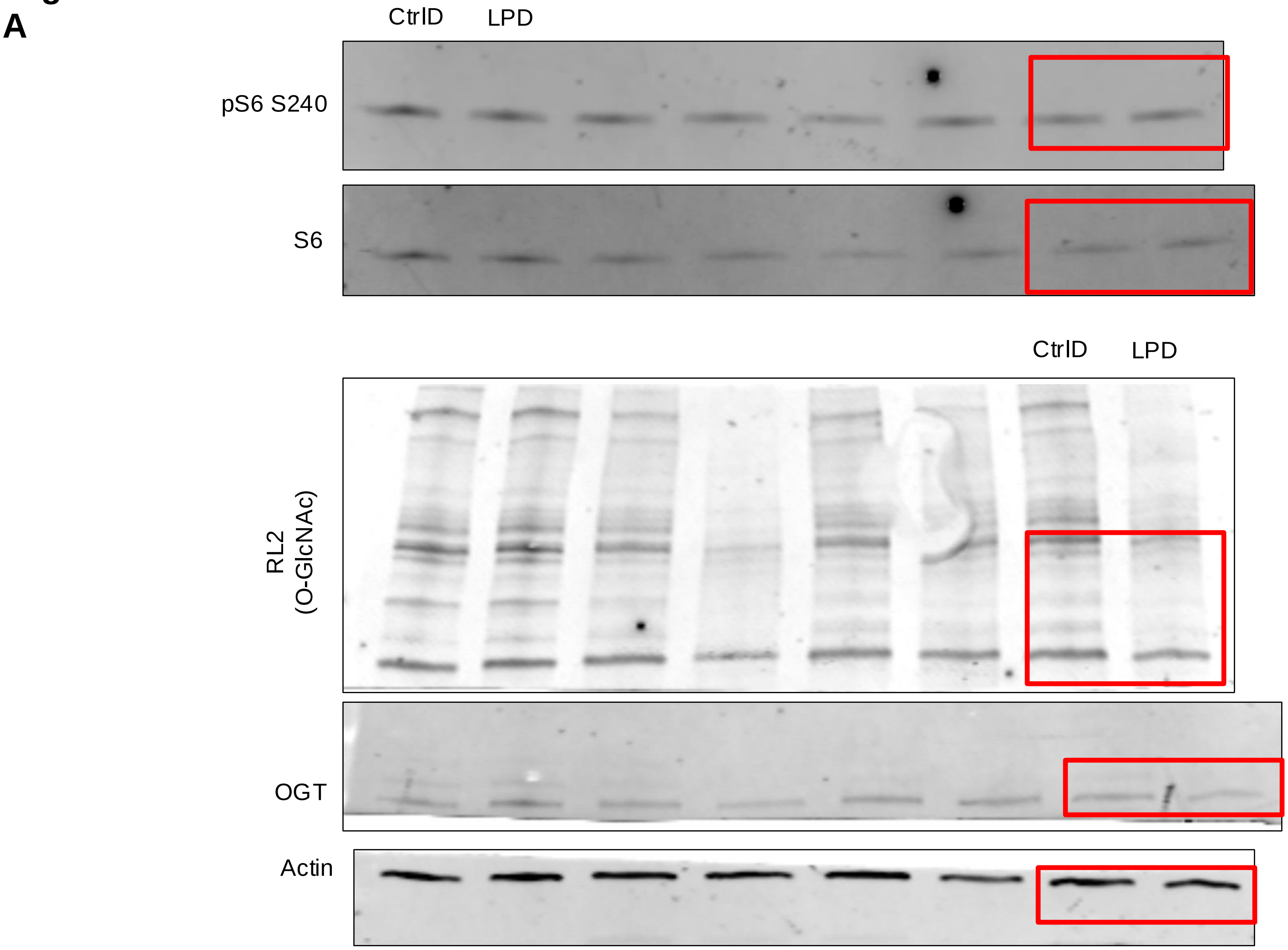

Figure 1

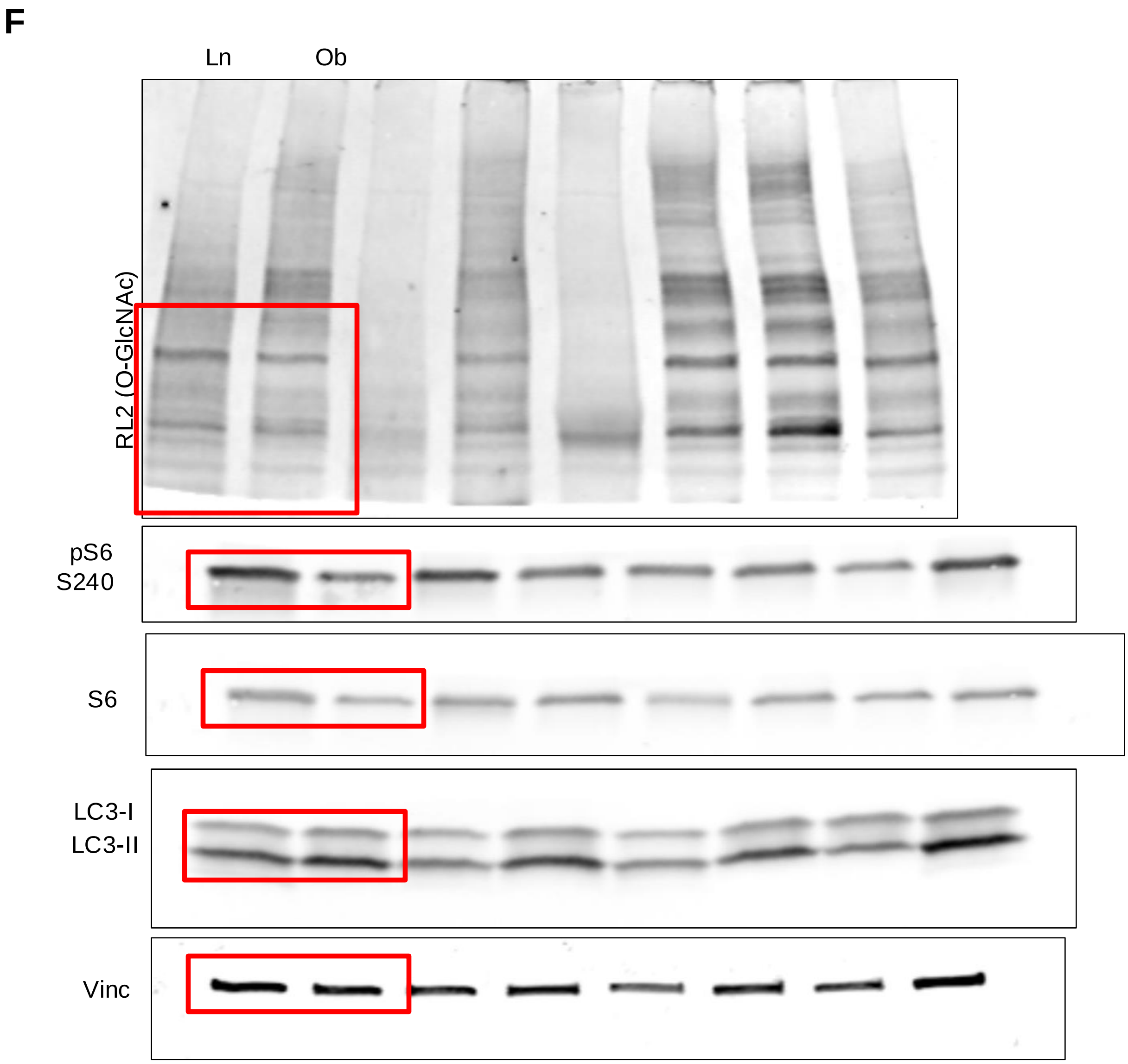

Figure 1

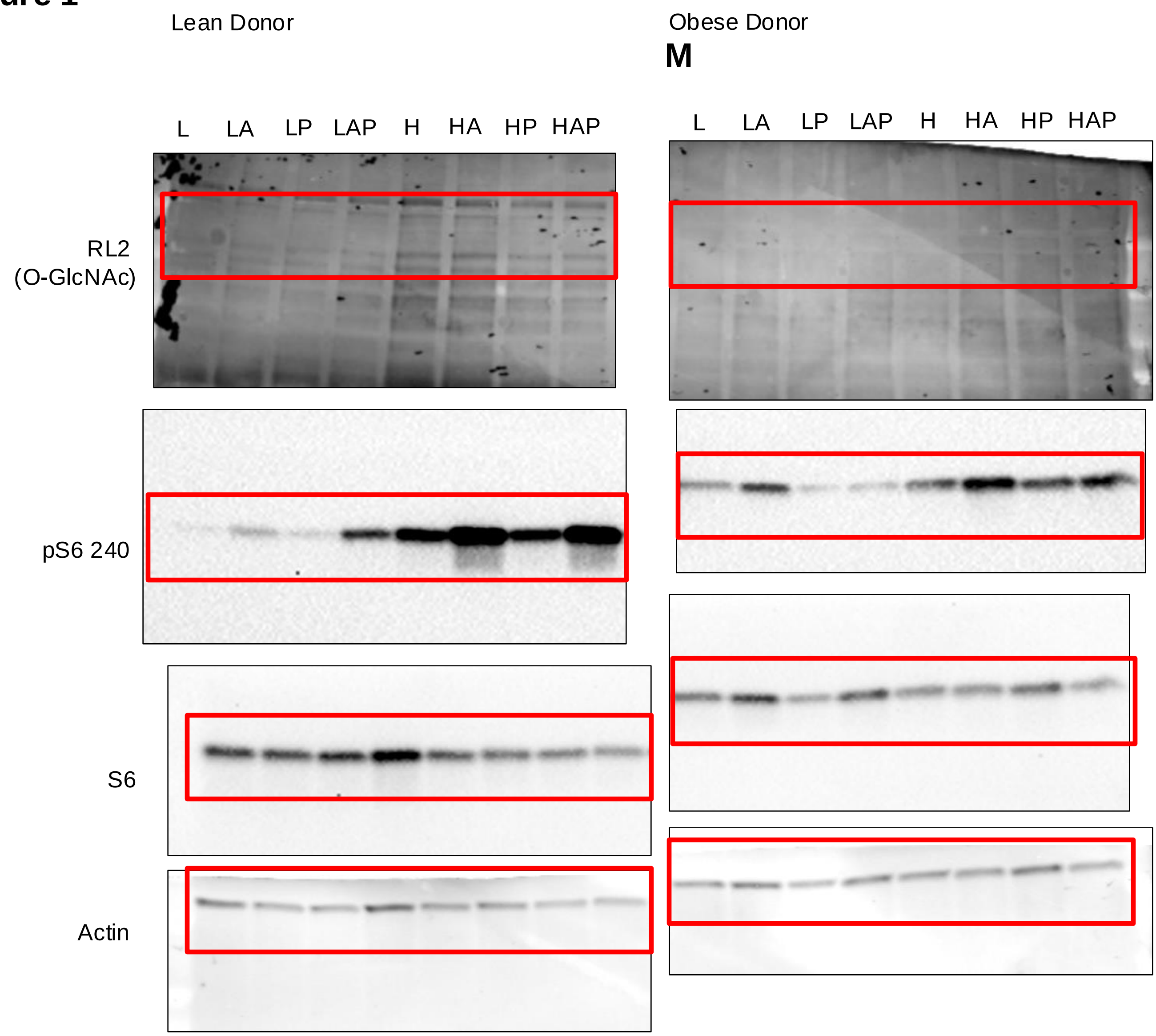
Lean Donor
Obese Donor
M
L
LA
LP
LAP
H
HA
HP
HAP
L
LA
LP
LAP
H
HA
HP
HAP
RL2
(O-GlcNAc)
pS6 240
S6
Actin

Figure 2

B

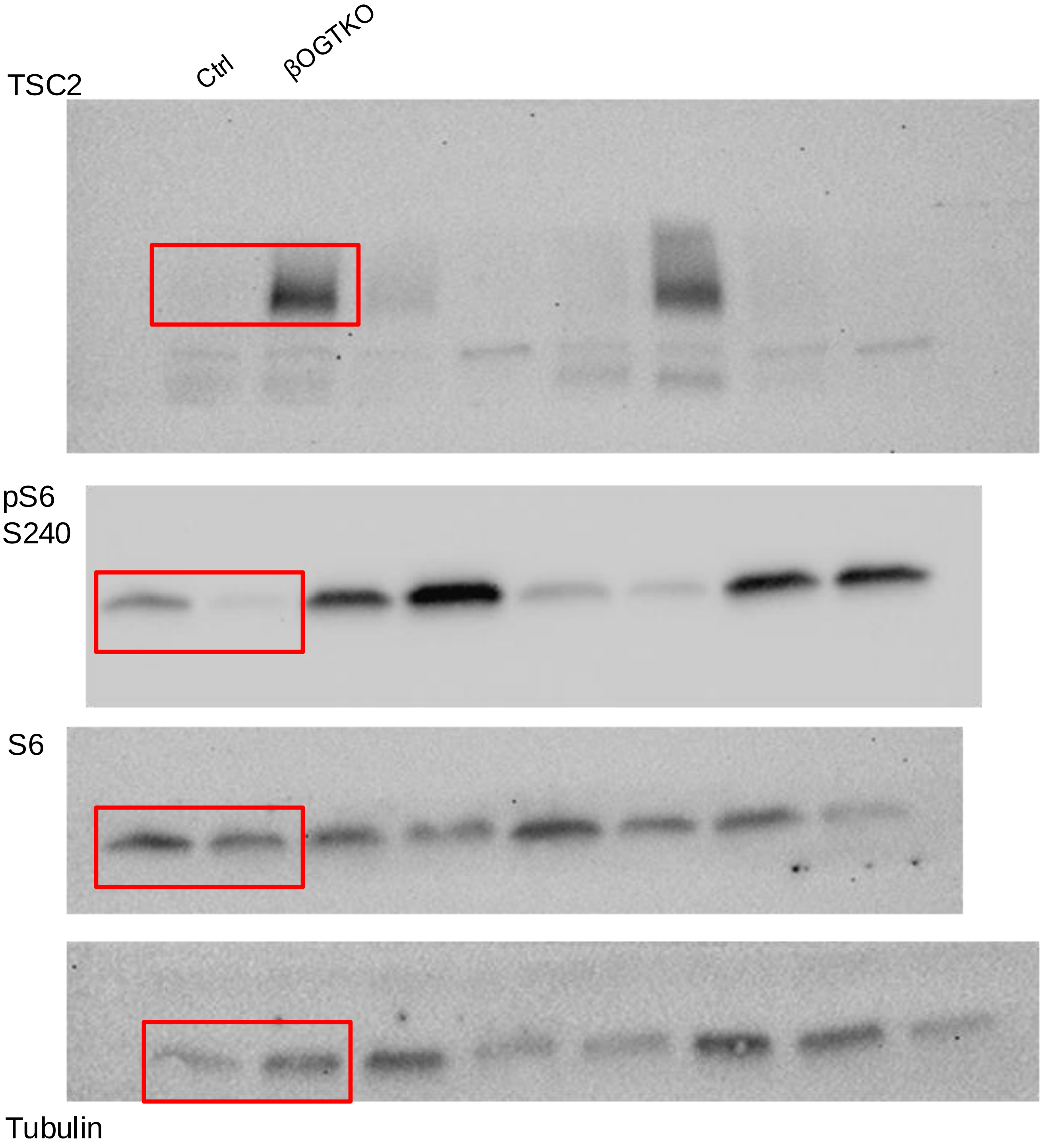

Figure 2

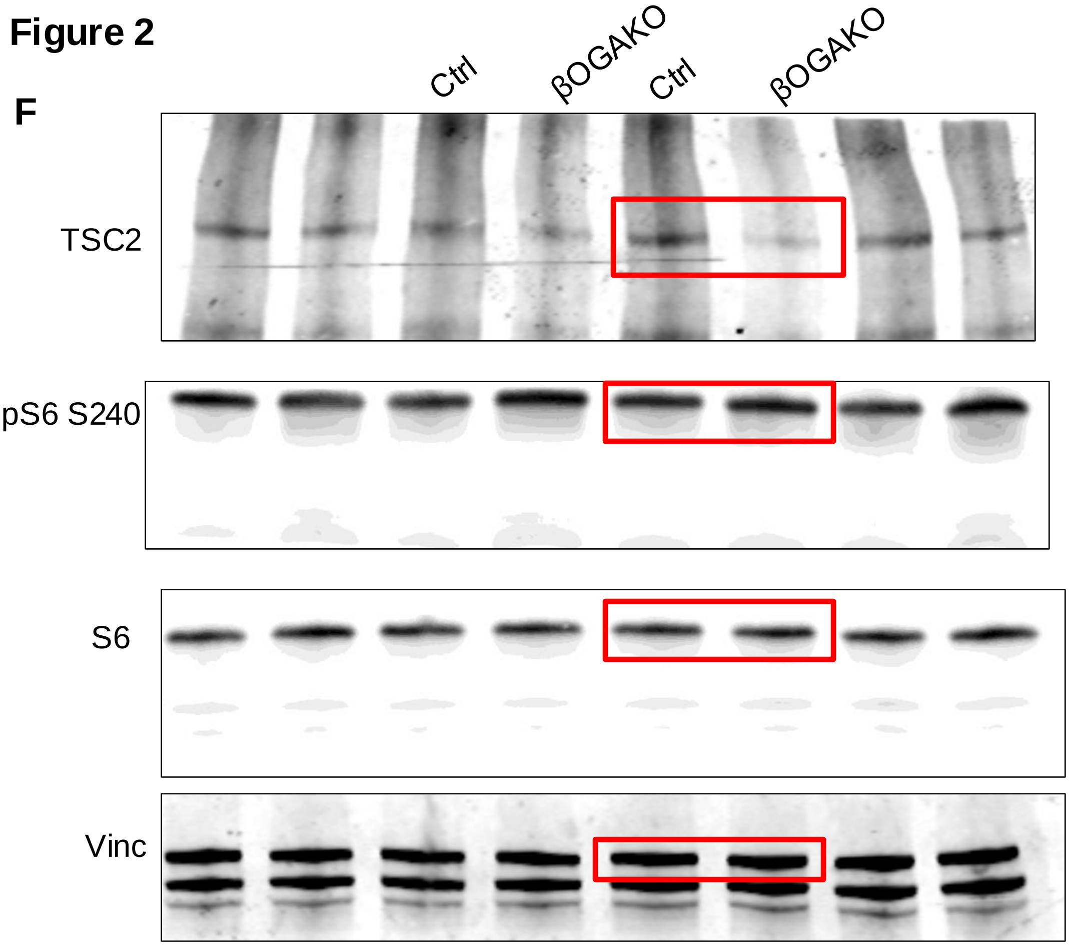

Figure 2

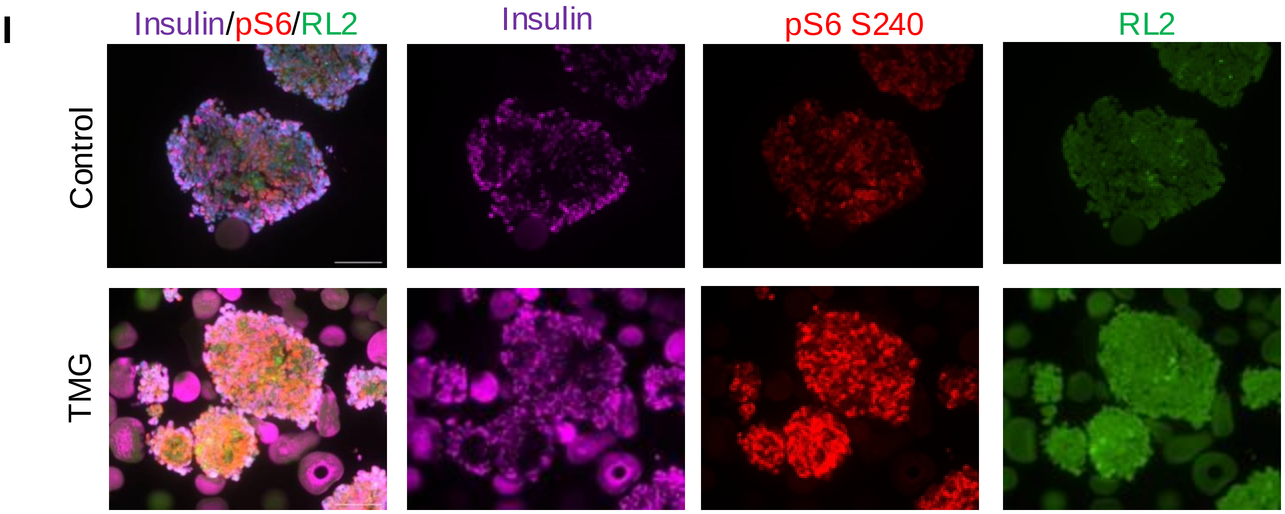

Figure 3

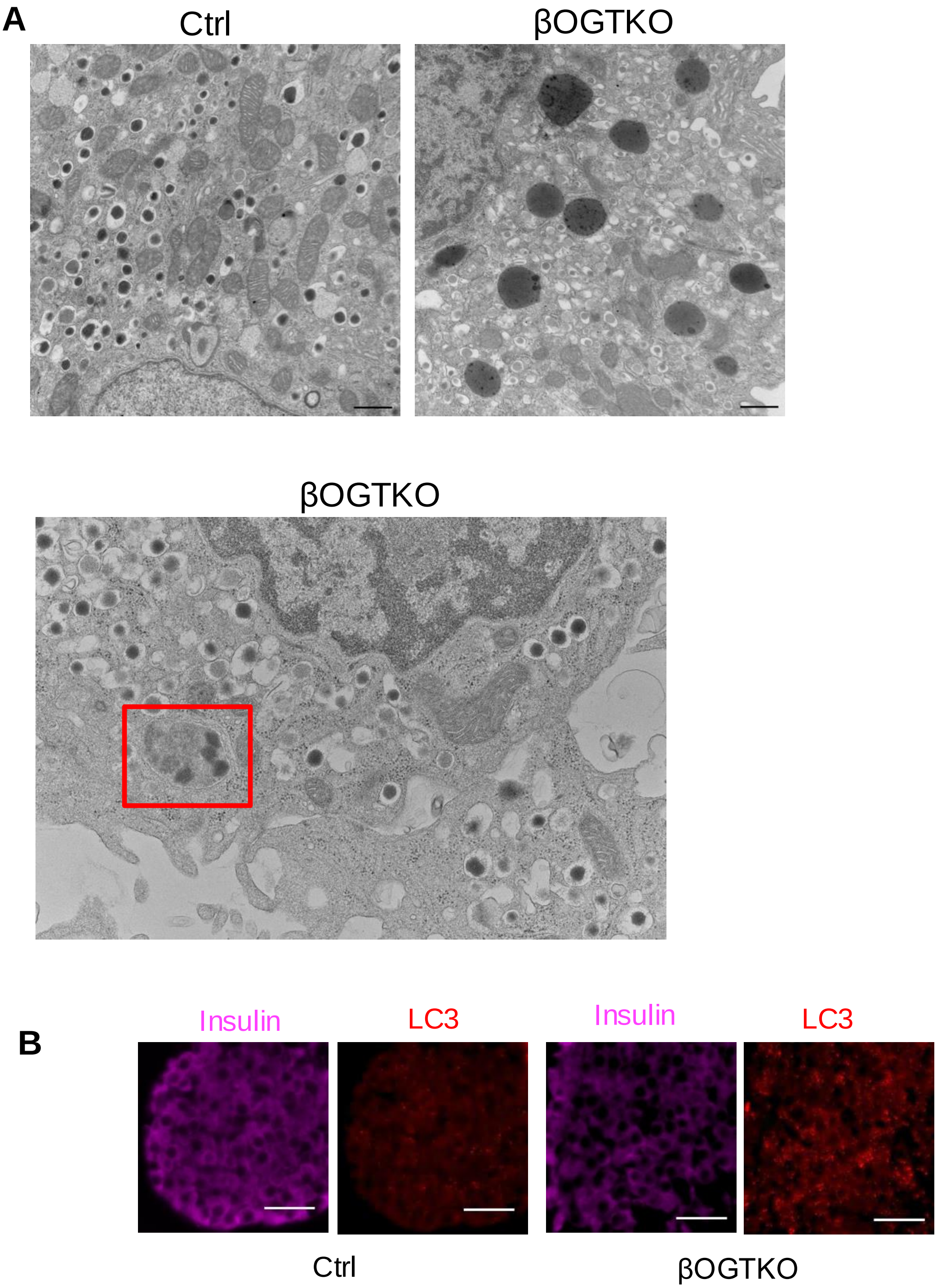

Figure 3

D

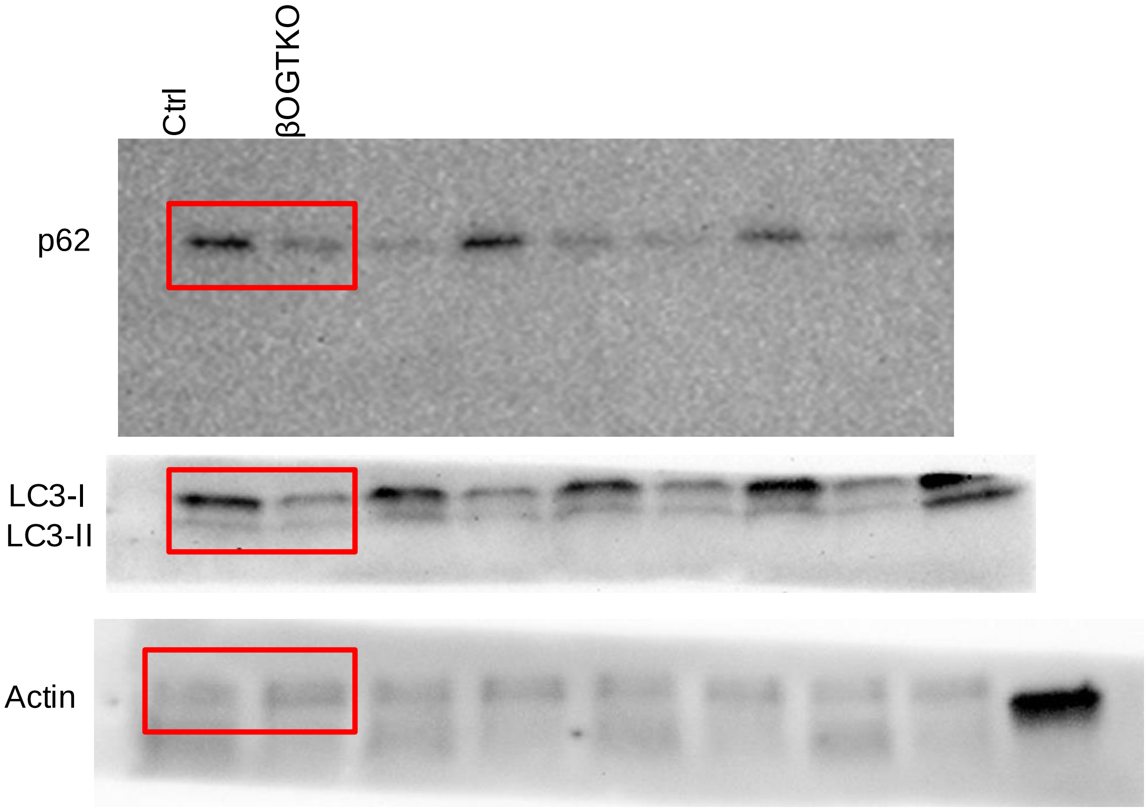

Ctrl
βOGTKO
p62
LC3-I
LC3-II
Actin

G

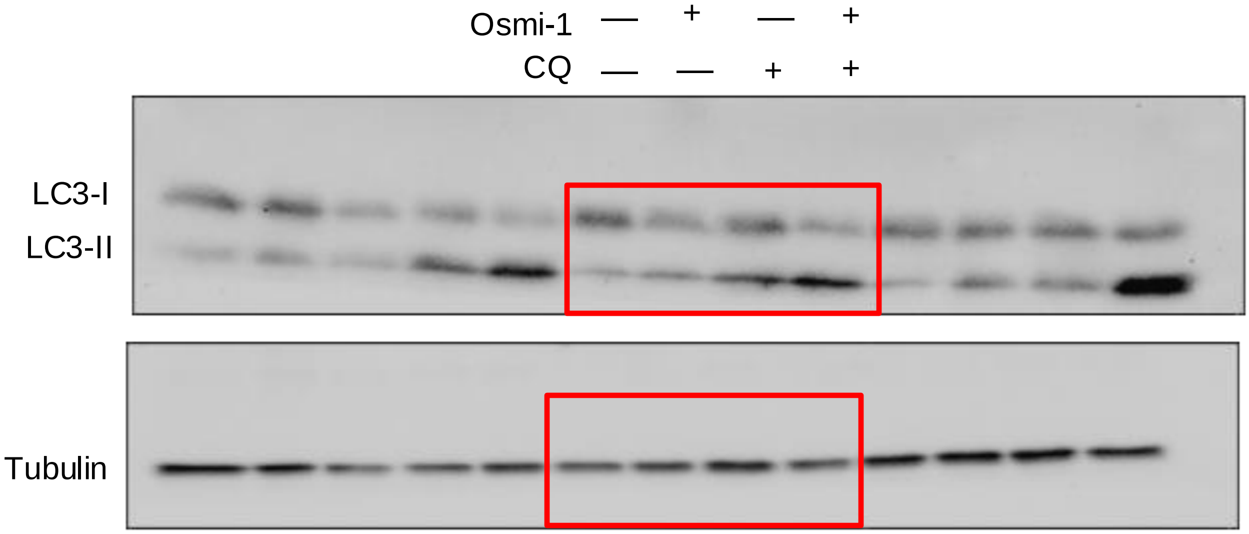

Osmi-1 — + — +
CQ — — + +
LC3-I
LC3-II
Tubulin

Figure 3

H

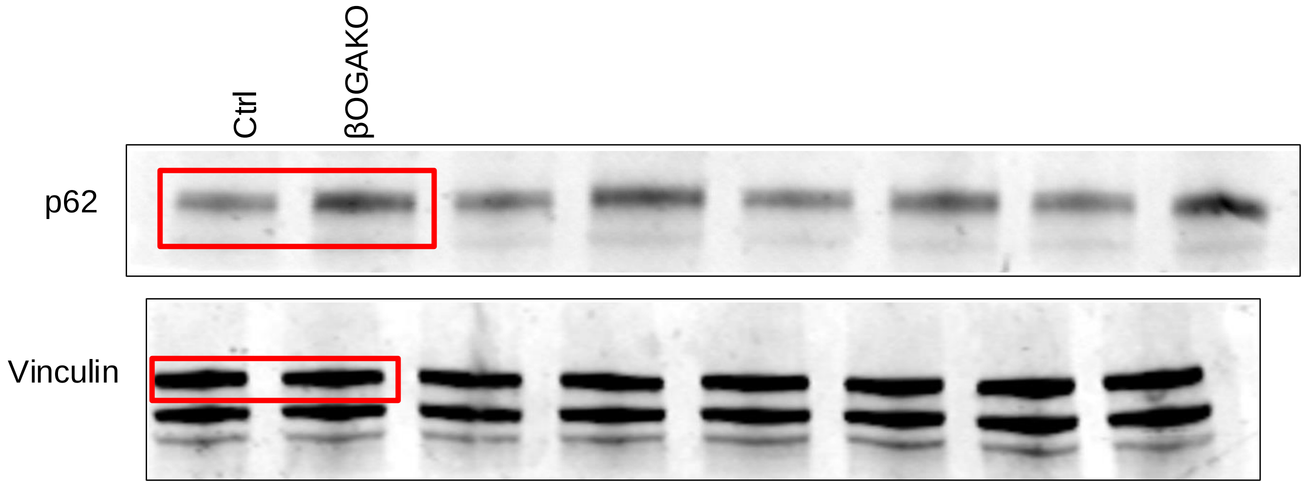

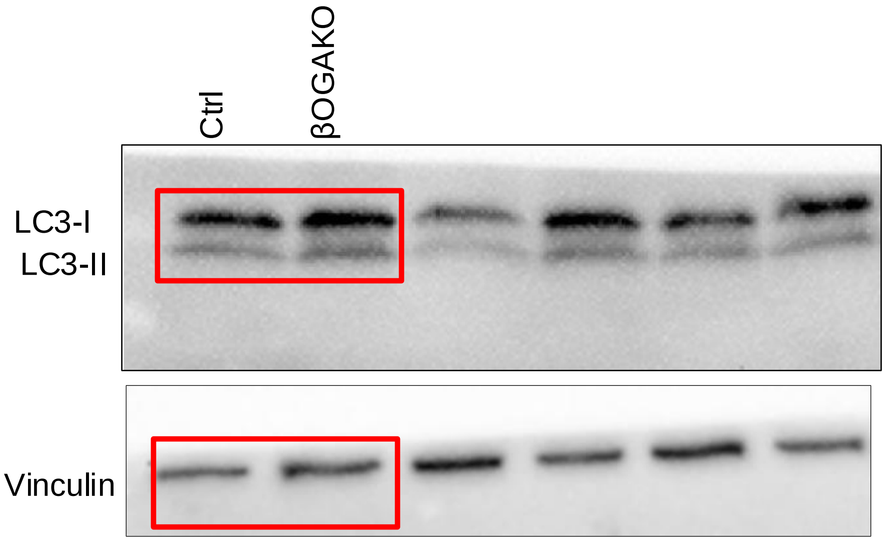

Figure 5

A

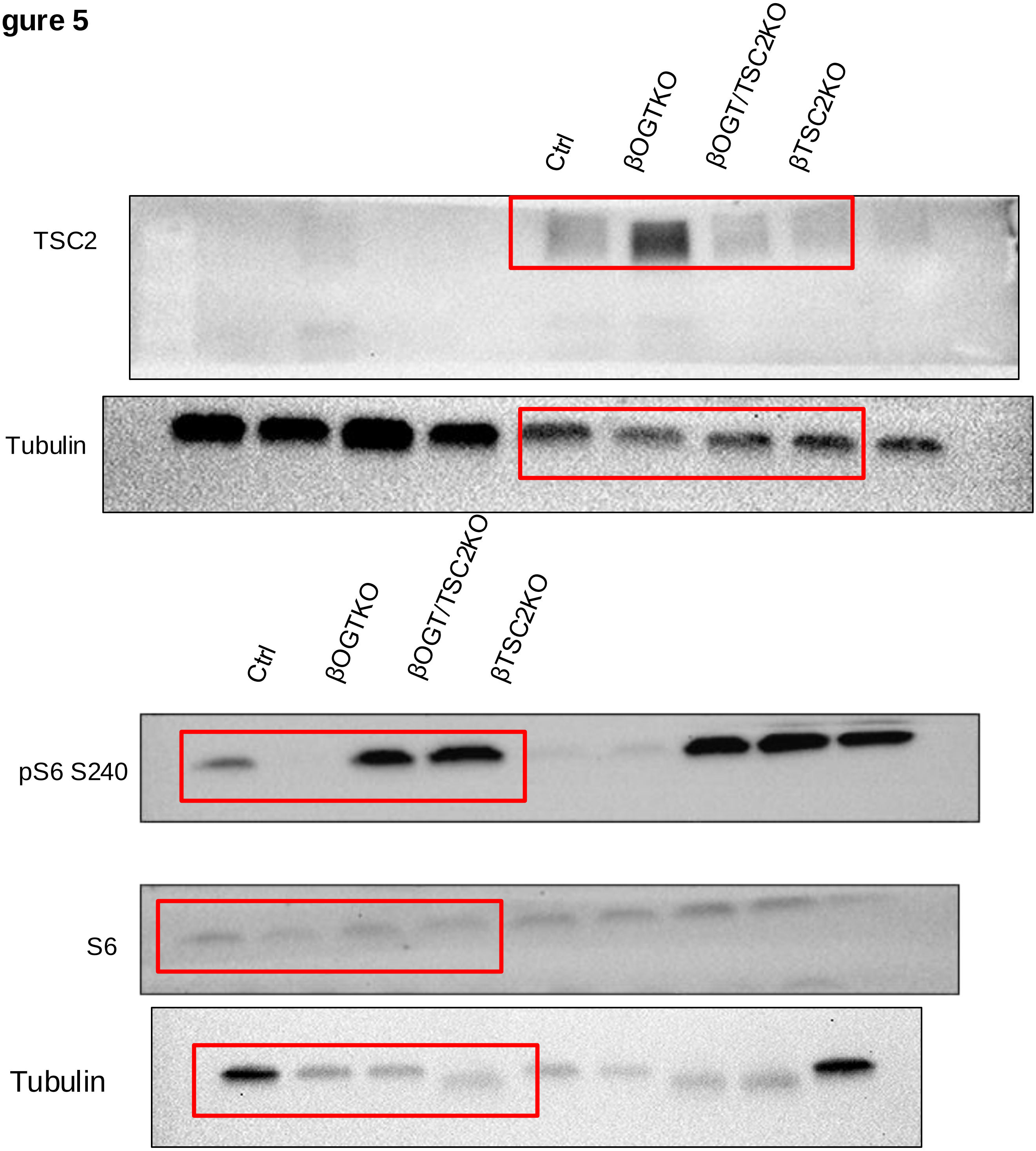
Ctrl
βOGTKO
βOGT/TSC2KO
βTSC2KO
TSC2
Tubulin
Ctrl
βOGTKO
βOGT/TSC2KO
βTSC2KO
pS6 S240
S6
Tubulin

Figure 6

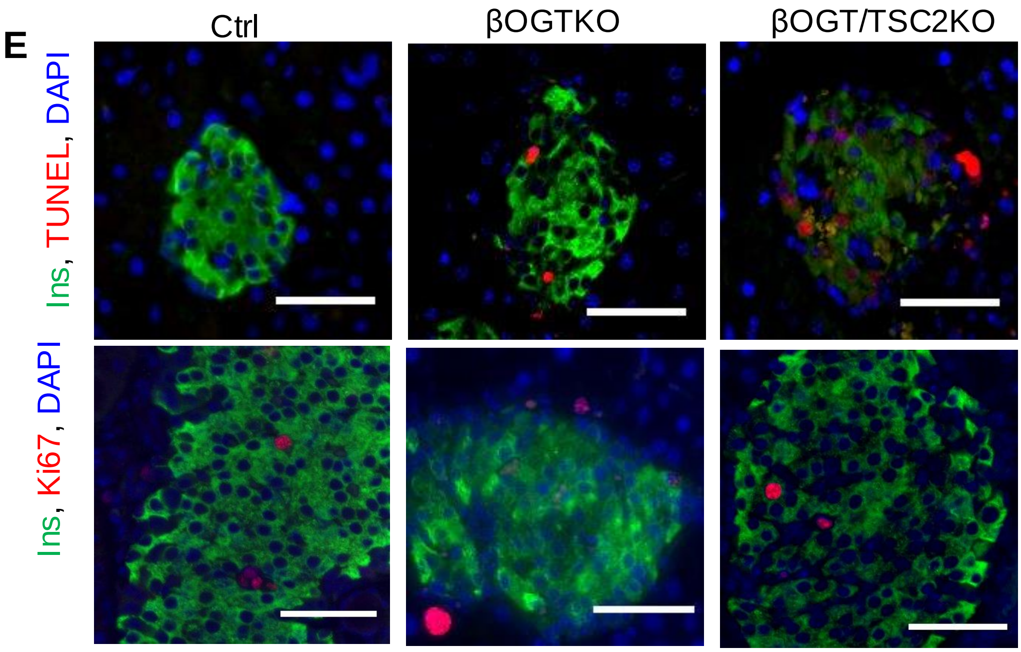

Supplemental Figure 1

A

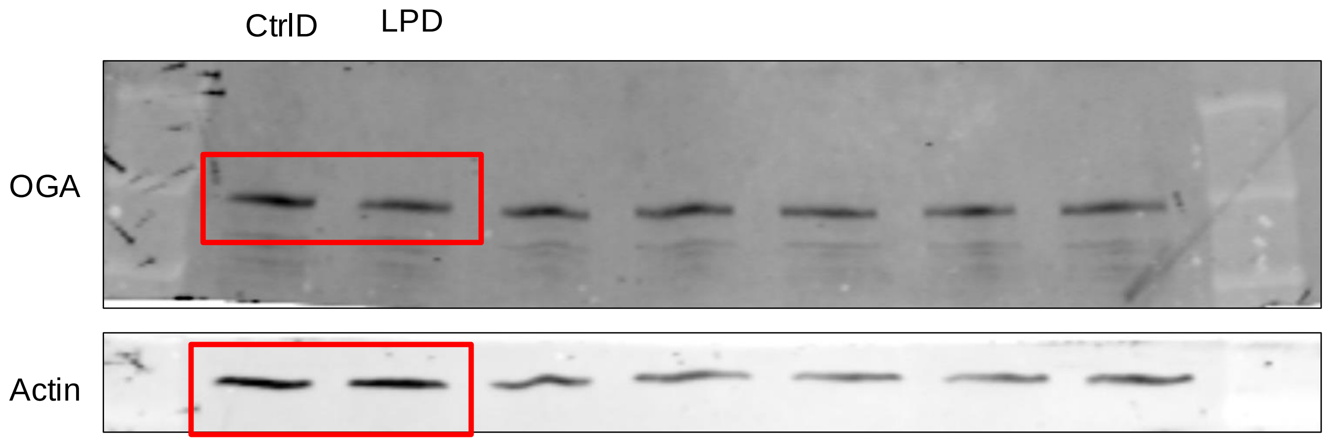

D

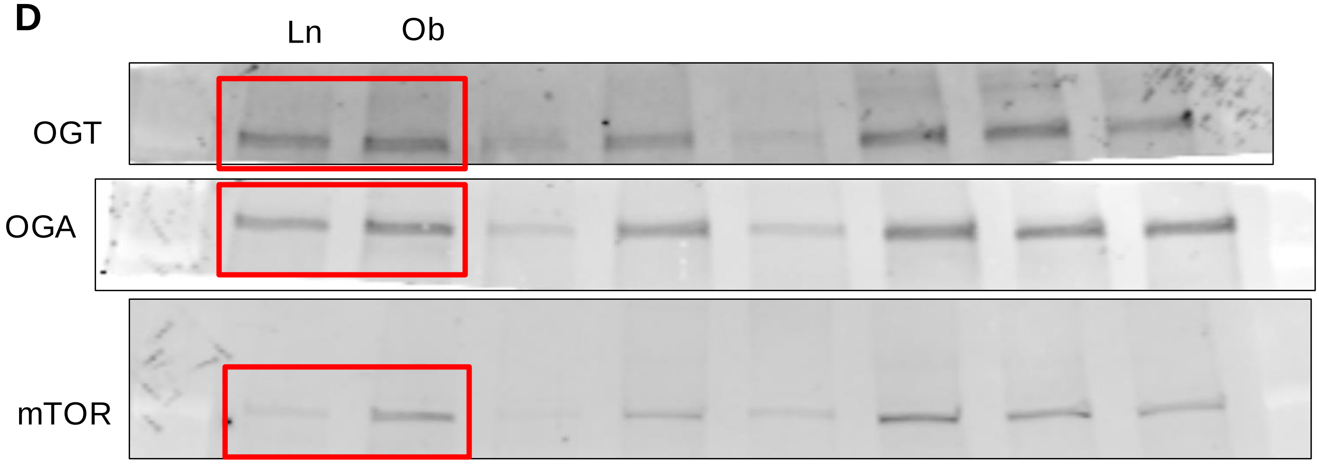

Supplemental Figure 2

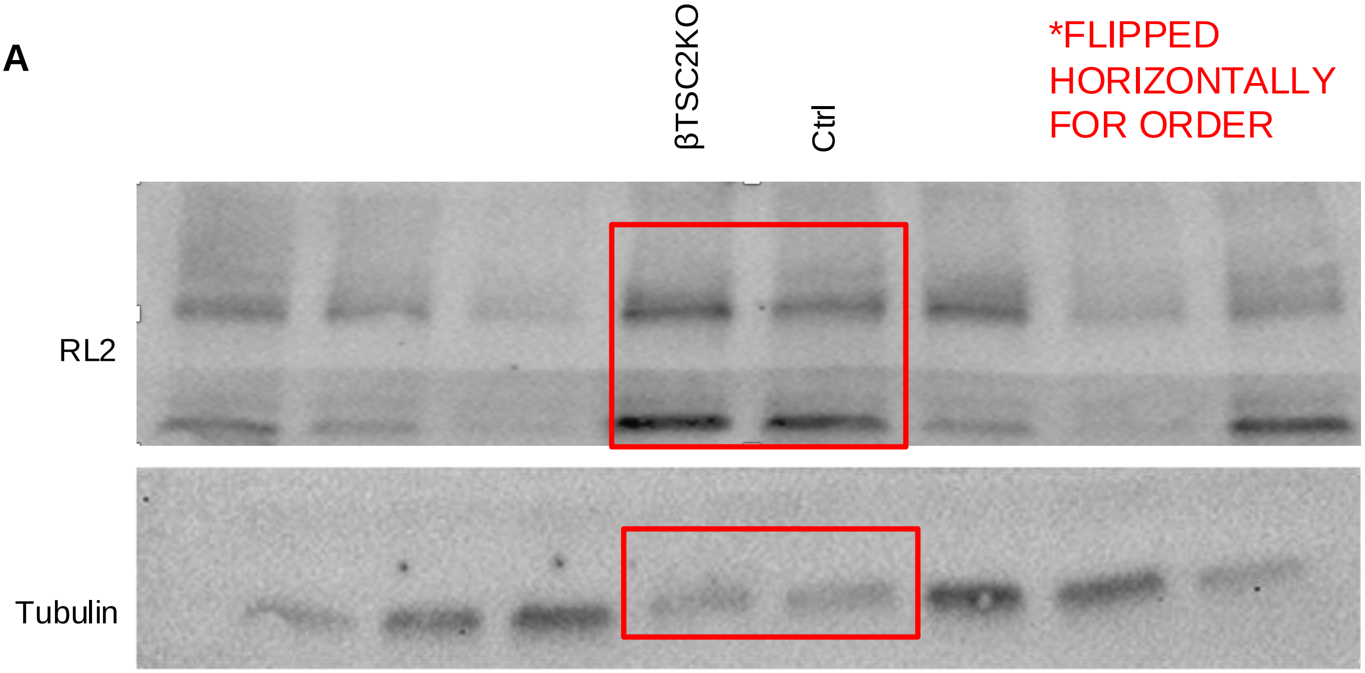

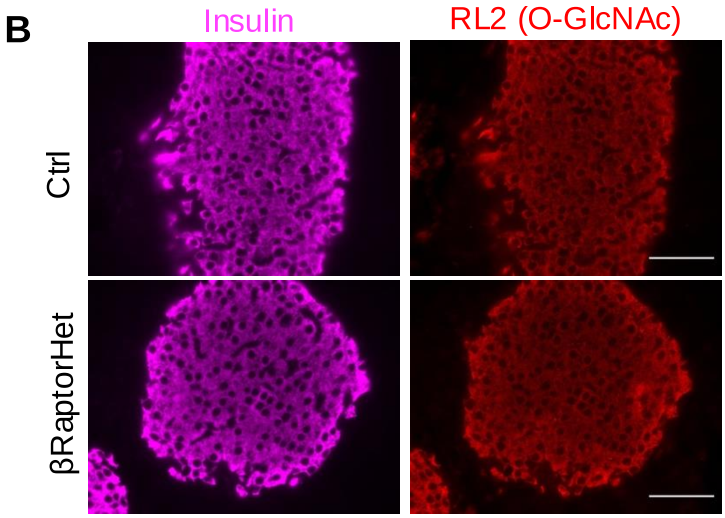

Supplemental Figure 5

B

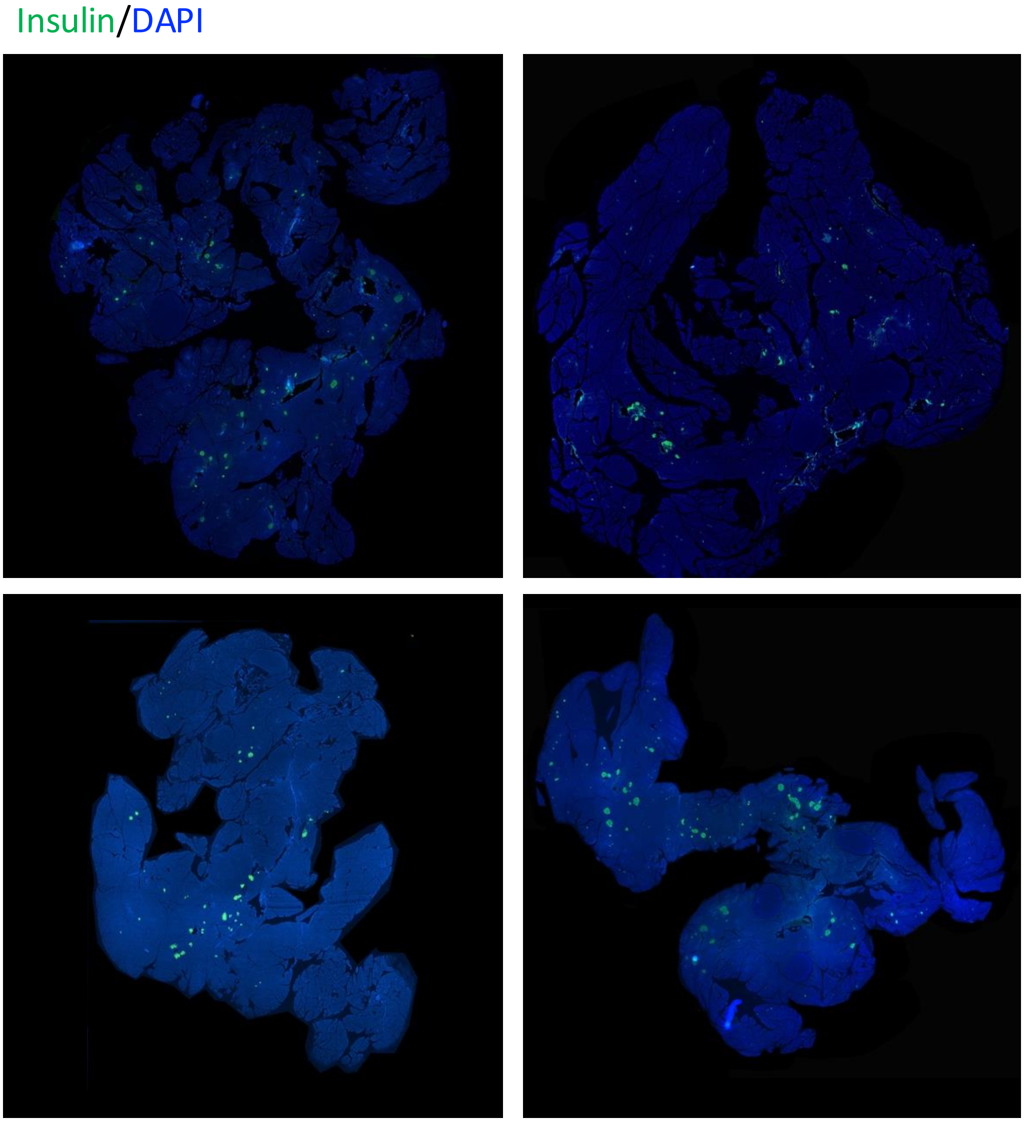

Supplement: Unedited blot and gel images [file jciinsight-9-183033-s229.pdf]
